# Respiratory Heme A-Containing Oxidases Originated in the Ancestors of Iron-Oxidizing Bacteria

**DOI:** 10.3389/fmicb.2021.664216

**Published:** 2021-06-15

**Authors:** Mauro Degli Esposti, Ana Moya-Beltrán, Raquel Quatrini, Lars Hederstedt

**Affiliations:** ^1^Center for Genomic Sciences, Universidad Nacional Autónoma de México (UNAM), Cuernavaca, Mexico; ^2^Fundación Ciencia & Vida, Santiago, Chile; ^3^ANID–Millennium Science Initiative Program–Millennium Nucleus in the Biology of the Intestinal Microbiota, Santiago, Chile; ^4^Facultad de Medicina y Ciencia, Universidad San Sebastian, Santiago, Chile; ^5^The Microbiology Group, Department of Biology, Lund University, Lund, Sweden

**Keywords:** cytochrome oxidase, heme A synthase, CtaA, CtaG, bacterial evolution

## Abstract

Respiration is a major trait shaping the biology of many environments. Cytochrome oxidase containing heme A (COX) is a common terminal oxidase in aerobic bacteria and is the only one in mammalian mitochondria. The synthesis of heme A is catalyzed by heme A synthase (CtaA/Cox15), an enzyme that most likely coevolved with COX. The evolutionary origin of COX in bacteria has remained unknown. Using extensive sequence and phylogenetic analysis, we show that the ancestral type of heme A synthases is present in iron-oxidizing Proteobacteria such as *Acidithiobacillus* spp. These bacteria also contain a deep branching form of the major COX subunit (COX1) and an ancestral variant of CtaG, a protein that is specifically required for COX biogenesis. Our work thus suggests that the ancestors of extant iron-oxidizers were the first to evolve COX. Consistent with this conclusion, acidophilic iron-oxidizing prokaryotes lived on emerged land around the time for which there is the earliest geochemical evidence of aerobic respiration on earth. Hence, ecological niches of iron oxidation have apparently promoted the evolution of aerobic respiration.

## Introduction

Aerobic respiring organisms contain terminal oxygen reductases for energy metabolism at different oxygen levels in the environment. The evolutionary origin of these oxygen-consuming enzymes is unknown (Castresana and Saraste, 1995; Pereira et al., 2001; Han et al., 2011; Sharma and Wikström, 2014). A common class of terminal oxidases is heme A-containing proton pumping cytochrome oxidase (COX), which has a relatively low affinity for oxygen (Han et al., 2011; Degli Esposti et al., 2019) and consequently must have evolved during or after the Great Oxygenation Event (GOE), which produced stable levels of oxygen on earth (Konhauser et al., 2011). COX of the family A of Heme Copper Oxygen Reductases (HCO) is widespread in all kingdoms of life (Castresana and Saraste, 1995; Pereira et al., 2001; Han et al., 2011; Degli Esposti, 2020) due to extensive Lateral Gene Transfer (LGT) (Nelson-Sathi et al., 2015; Soo et al., 2017). Subsequently bacterial COX became the cytochrome *c* oxidase of mitochondrial organelles. The prokaryotes that initially evolved COX have remained elusive (Pereira et al., 2001; Soo et al., 2017; Degli Esposti et al., 2019; Matheus Carnevali et al., 2019) and, consequently, the origin of aerobic respiration is a major unresolved problem. To address the question of COX origin, we have studied the phylogeny of two proteins specifically required for COX biosynthesis and performed new and expanded analyses on the phylogeny of the major protein subunit of COX. The latter analysis is focused on family A oxidases since the phylogenetic distribution of family B oxidases, which also have heme A, is rather patchy among bacteria. Indeed, it is possible that family B derived from some ancestral family A oxidase (Degli Esposti, 2020). Combined with other findings, our results suggest that family A COX has evolved in ancestors of extant acidophilic iron-oxidizing bacteria.

**FIGURE 1:**
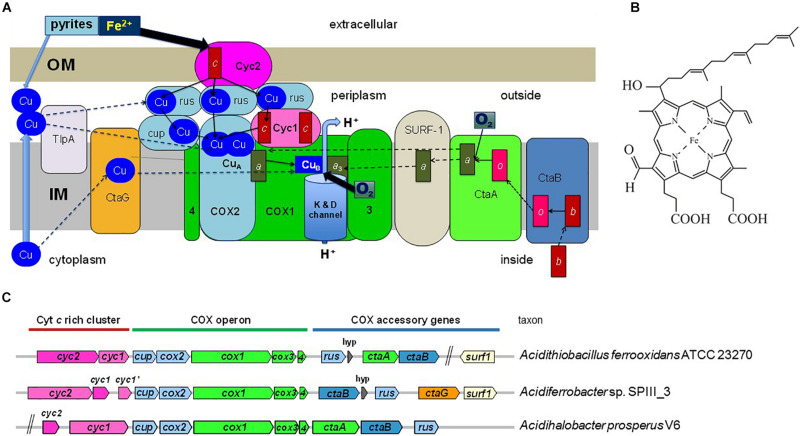
Energy metabolism and COX gene clusters of iron-oxidizing bacteria. **(A)** The figure illustrates the electron transport system in the cytoplasmic membrane of iron-oxidizing bacteria and proteins that function in COX biogenesis. The two heme A molecules in the COX1 subunit are indicated by dark green rectangles labeled *a* and *a*_3_. COX2 and other Cu-binding proteins are colored in pale blue, while proteins containing *c*-type cytochromes (heme C is indicated by the red rectangle) are in dark pink. COX3 and COX4 are abbreviated as 3 and 4, respectively, and are colored in bright green as subunit COX1. OM and IM indicate the outer and inner (cytoplasmic) membrane of the bacterium. Rusticyanin (rus) is shown in multiple copies because it is present in large excess with respect to the other redox proteins of the system (Blake et al., 2016). Cup refers to membrane-bound cupredoxin proteins (Degli Esposti, 2020). TlpA stands for thioredoxin-like protein A, a membrane-bound protein involved in Cu_A_ assembly. Thin arrows indicate electron transfer routes while dashed arrows indicate steps in COX biogenesis. The water enzyme product of COX is not shown. The overall scheme of electron transfer from extracellular donors to oxygen reduced in the cytoplasmic membrane applies to a variety of bacteria that have multiheme *c* cytochromes instead of rusticyanin mediators (Deng et al., 2018). **(B)** Structure of heme A. The difference of this variant of heme compared to heme B (protoheme IX) is the hydroxyethyl-farnesyl group and the formyl group. **(C)** Gene clusters for COX in bacteria often contain genes for heme A synthesis (*ctaA, ctaB*) and COX assembly (*ctaG, surf1*), as illustrated by representative gene clusters of iron-oxidizing Proteobacterial taxa. The size of the gene symbols is roughly proportional to gene length. hyp, gene for a short hypothetical protein.

COX in mitochondria and aerobic bacteria is a multi-protein intrinsic membrane complex with several metal prosthetic groups. Subunit I, COX1, contains two heme A molecules (Figures 1A,B), denoted cytochrome *a* and *a*_3_ in the assembled enzyme, and one copper atom, Cu_B_. COX2 contains two copper atoms in a binuclear center, Cu_A_ (Figure 1A). The redox reaction of molecular oxygen reduction at the heme *a*_3_-Cu_B_ center is coupled to the generation of an electrochemical gradient via conserved proton-conducting channels (Pereira et al., 2001; Ferguson and Ingledew, 2008; Han et al., 2011; Sharma and Wikström, 2014; Degli Esposti, 2020). This gradient drives ATP synthesis and various other energy-demanding functions in the cell.

Biosynthesis of COX requires multiple proteins that catalyze the formation of heme A, or are involved in the insertion of the metal cofactors and overall assembly of the enzyme in the membrane (Figure 1A). Heme A as a prosthetic group is only found in respiratory oxygen reductases (Hederstedt, 2012). The biosynthesis of heme A from protoheme IX (heme B) involves two enzymes (Figure 1A; Mogi et al., 1994; Hederstedt, 2012). First heme O synthase, CtaB/Cox10/CyoE, catalyzes formation of heme O and then heme A synthase, CtaA/Cox15, converts heme O into heme A. Synthesis of heme A Figure 1B requires ambient oxygen levels (Brown et al., 2002) that were attained in primordial earth only after the GOE. In bacteria, the genes for COX proteins and those for heme A synthesis and COX assembly factors show modularity, i.e., the genes are frequently clustered in the chromosome (Figure 1C). Based on the assumption that COX proteins and protein factors specifically required for COX biosynthesis have coevolved, we analyzed the phylogeny of bacterial CtaA to obtain insights on how COX has evolved. The results prompted us to establish a revised expanded classification of heme A synthases and develop a scheme for their evolution based upon phylogenetic data, which was compared to that of COX1 and the COX-specific assembly factor CtaG to define the likely origin of COX.

## Materials and Methods

### Phylogenetic Analysis

Database searches and genome scanning were conducted by iterative BlastP (Basic Local Alignment Search Tool for Proteins) searches as detailed in the Supplementary Material. Briefly, wide searches expanded to 5000 hits were usually performed with the DELTABlast program using the BLOSUM62 substitution matrix (Boratyn et al., 2012). Integrated searches were expanded in granular detail to recognize established conserved domains of the (super) family to which it may belong (as shown in the NCBI protein website)^1^ (Lu et al., 2020) preferentially using BlastP and PSI-BLAST searches restricted to 500 hits. Classification of HCO has been undertaken using the bioinformatic classifier available at http://www.evocell.org/hcoevocell.org (Sousa et al., 2011).

Maximum likelihood (ML) trees were initially produced with the MEGA5.2 program to elaborate BLAST outputs and preliminary phylogenetic frameworks (Degli Esposti et al., 2020), with a variety of substitution models (generally the WAG model for multitopic membrane proteins and the Dayhoff or BLOSUM62 substitution matrix for membrane-anchored, predominantly water-exposed proteins, and discrete gamma distribution of four categories to account for evolutionary rate differences among sites and lineages, allowing some sites to be evolutionarily invariable (Tamura et al., 2011). The MEGA program was also used to transform ML trees into ultrametricized trees similar to BEAST tree outputs using the condensed option, with a cut-off of 50% bootstrap support.

Phylogenetic inference was routinely undertaken using either Bayesian or ML probabilistic approaches. We reconstructed ML trees using the more sophisticated IQ-Tree program (Nguyen et al., 2015), normally from its server http://iqtree.cibiv.univie.ac.at/ (Trifinopoulos et al., 2016) with the ultrafast bootstrap option of 1000 replicates and the LG model as in previous publications (Degli Esposti et al., 2019; Spang et al., 2019). Additionally, we used mixture models of amino acid substitution, such as C20 (Le et al., 2008) and EX_EHO (Le and Gascuel, 2010). We found that the EX_EHO model tended to produce trees with stronger support than those obtained with the LG model for CtaA and COX1 proteins, but not with caa3_CtaG proteins, probably because of their limited conservation. ML trees were reconstructed also with the program PhyML 3.0 (Guindon et al., 2010), and generally run from the online platform http://www.phylogeny.fr/index.cgi using the WAG model and the Shimodaira–Hasegawa (SH)-like statistical support analysis. We used the program FigTree 1.4.4^2^ for visualizing trees obtained with various methods.

We preferentially used the BEAST 2.6.2 package for Bayesian phylogenetic inference (Drummond et al., 2006) because of its information-rich outputs (Lee et al., 2013; Drummond and Bouckaert, 2015). Although predominantly utilized for divergence studies of animal species with genes including mitochondrial COX1-3 (Lee et al., 2013; Ratnasingham and Hebert, 2013), BEAST analysis has been applied also to study the molecular evolution of bacterial redox proteins (Khadka et al., 2018; Bouckaert et al., 2019). Manually curated alignments were first loaded into the BEAUti 2 app of the package to prepare.xml files containing the detailed settings for the phylogenetic analysis run with the BEAST program (Bouckaert et al., 2019). Routinely, such settings included: four gamma categories with shape 0.3 (or other empirically estimated values) and proportion of invariant sites from 0.01 to 0.1, depending on the protein and its taxonomic sampling; BLOSUM62 or WAG as substitution models; Relaxed Clock Log model and the gamma option for the Yule birth model (other priors were left in their default setting); at least 2 million iterations for the Markov chain of Monte Carlo analysis with 5 pre-burnin. Trees were stored every 1000 iterations and then usually reduced by 10% burnin to generate Maximal Clade Credibility (MCC) output files with minimal posterior value of 0.1 using the TreeAnnotator app (Drummond and Bouckaert, 2015). MCC trees were graphically elaborated with the FigTree program, which produced a wealth of quantitative data for the various branches (Lee et al., 2013; Khadka et al., 2018), or visualized using the program DensiTree 2 (Bouckaert and Heled, 2014; Drummond and Bouckaert, 2015). In a few cases, we also used the program MrBayes v.3.0b4 as in reference (Degli Esposti et al., 2020).

### Protein Sequence Analysis

Given that the quality of phylogenetic trees heavily depends upon the accuracy of the protein sequence alignments upon which they are reconstructed, we performed an in depth analysis of the sequence variation of each protein to guide its proper alignment. This analysis was undertaken by exploiting the versatility of the MEGA programs after importing whole sequences or alignments of multiple sequences downloaded directly from the BlastP searches. An initial alignment of the protein was built with a minimal set of 20 sequences using either the ClustalW or the MUSCLE algorithm within the MEGA5 program (Tamura et al., 2011). The alignments thus obtained contained several gaps that were often unnecessary to maximize for local sequence similarity, as verified by visual inspection; such gaps were then removed along manual refinements conducted with iterative rounds of implementation that were aided by the inclusion, whenever possible, of protein sequences for which 3D information is currently available. The alignments were then progressively enlarged to include sequences that were representative of different prokaryotic taxa in which the protein was found, with additional refinements to accommodate local sequence variations. Short residue gaps that were needed to properly align a single sequence were subsequently deleted along detailed manual refinements of local sequence similarity and congruent hydropathy profile.

The enlarged alignments thus refined were used to build phylogenetic trees encompassing most major molecular variants, as well as the overall taxonomic distribution of any protein studied. Some of the alignments are available upon request. Sequences that produced long branches or displayed high substitution rates were identified by statistical analysis as described in Supplementary Material. Such sequences were subsequently removed, often substituted by sequences clustering in the same subclade that did not display equivalent branch aberrations. Then the set of aligned sequences for a given protein was reduced to simplify tree presentation without altering the tree topology found with larger alignments.

The CtaA, caa3_CtaG and COX1 proteins studied here have multiple membrane-spanning segments (TM). Consequently, we have applied extensive hydropathy analysis to all proteins analyzed, using both the TMpred server https://embnet.vital-it.ch/software/TMPRED_form.html and the TMHMM Server v. 2.0 https://hsls.pitt.edu/obrc/index.php?page=URL1164644151. These programs utilize complementary methodologies that help define the ends of predicted TM (Möller et al., 2001). This sequence analysis was combined with the available 3D structural information for COX subunits (Iwata et al., 1995; Svensson-Ek et al., 2002), and CtaA (Niwa et al., 2018), to define the TM regions and other topological features in distant protein homologs, as in the case of proteins from iron-oxidizers. Membrane topology was graphically rendered with the program TOPO2^3^ and then used as a platform for building the protein models.

**FIGURE 2:**
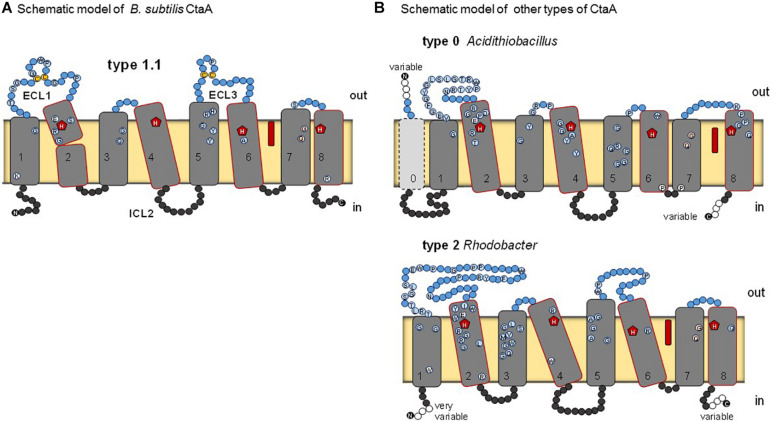
Schematic models for various types of heme A synthase. **(A)** *B. subtilis* CtaA, based on the reported crystal structure of the protein (Niwa et al., 2018). Residues conserved among type 1.1 CtaA are shown by circles. ECL1 and ECL3 indicate extracellular loops on the periplasmic side (outer side) of the membrane. Heme B (red elongated symbol) occupies the cofactor domain. Disulfide linked cysteine residues in ECL1 and ECL3 are in yellow. **(B)** Models for type 0 and type 2 CtaA represented by the *Acidithiobacillus ferrooxidans* and *Rhodobacter capsulatus* enzymes, respectively. See Table 1 for the classification of CtaA proteins. Residues that are conserved in the sequences of the represented types are shown by circles as in Panel **(A)**.

### Other Methods

Genome completeness was evaluated as described previously (Degli Esposti et al., 2019), or using information available in the GTNB database (Parks et al., 2018).

## Results and Discussion

### Iron-Oxidizing Bacteria Appear to Have an Ancestral Form of Heme A Synthase

Heme A synthase is required for the biosynthesis of the characteristic heme prosthetic groups in COX. The heme A synthase enzyme protein belongs to the super-family of Cox15-CtaA, cl19388, members of which are widespread among prokaryotes (He et al., 2016). The 3D structure of *Bacillus subtilis* CtaA has been determined by X-ray diffraction crystallography (Niwa et al., 2018) and that of *Aquifex aeolicus* analyzed by cryogenic electron microscopy (Zeng et al., 2020). *B. subtilis* CtaA has 8 TM and two extended extracellular loops, ECL1 and ECL3, each of which contains a conserved pair of cysteine residues linked by a disulfide bond (Lewin and Hederstedt, 2016; Niwa et al., 2018; Figure 2A). Overall, the CtaA protein comprises two nearly superimposable 4-helical bundle domains, with the C-terminal domain binding a *b*-type heme group via two conserved histidine residues, while the N-terminal domain seems to contain the catalytic site for conversion of heme O into heme A (Figure 2).

Many members of the Cox15-CtaA superfamily lack cysteine pairs in extracellular loops, for example *Rhodobacter capsulatus* CtaA (Figure 2B) and the eukaryotic Cox15 homologs. These variants have been called type 2 to distinguish them from type 1 proteins represented by *B. subtilis* CtaA (He et al., 2016). In recent explorative work on CtaA sequences (Degli Esposti et al., 2020) we found more variants, as presented in the expanded classification system (Table 1) and in the unrooted phylogenetic tree of Supplementary Figure 1 (left). We also discovered a potential root for the phylogeny of CtaA in the Domain of Unknown Function 420 (DUF420) that has four TM. CtaM of *Staphylococcus aureus*, *Bacillus anthracis* and *B. subtilis* contain the DUF420 domain and are required for assembly of active COX, but dispensable for heme A synthesis (Hammer et al., 2016; von Wachenfeldt et al., 2021).

**TABLE 1:** Classification of heme A synthase proteins.

Classification		Description					Taxonomic distribution
Class	Type	ECL1 length	Cys pair 1	ECL3 length	Cys pair 2	Other features	
D	0	Medium	No	Extremely short	No	Compact TM6&7	Acidithiobacilli, Fe-oxidizing gammaproteobacteria, *Nitrococcus, Metallibacterium*, *Salinisphaera*, *Defluviimonas*; thermoacidophilic Archaea: Thermoplasmatales, Sulfolobales and *Ca.* Marsarchaeota
C	1.0	Medium	Yes	Long	No	Long ICL2	Alicyclobacilli iron-oxidizers
C	1.0	Medium	Yes	Medium or short	No	Fused with ctaB or alone	Deinococus-Thermus, Chloroflexi MAG, Armatimonadetes, Verrucomicrobia, *Spirobacillus, Staphylococcus*, deltaproteobacteria, Euryarchaeaota MAGs
C	1.0	Long	Yes	Variable	No	Often substitution E57N *; many from metagenomes	Verrucomicrobia, CFB including *Ca.* Marinimicrobia, Balneolaeota, other phyla, unclassified bacteria
C	1.0	Medium or long	Yes	Medium or long	No	Cys pair 1 separated by 6 aa	Chloroflexi, Acetothermia (fused with CtaB)
C	1.0	Long	Yes	Very short	No	3D structure^, Cys pair 1 separated by 5 aa	Aquificae e.g., *Aquifex aeolicus* and *Hydrogenovirga*
B	1.1	Medium	Yes	Medium	Yes	3D structure∼, Cys pair 1 separated by 6 aa	Bacillales, Cyanobacteria, Actinobacteria, Proteobacteria, *Ktenobacter* (fused with CtaB)
B	1.1	Medium	Yes	Short	Yes	Substitution E57K and H123N *	betaproteobacteria e.g., *Ca.* Accumulibacter, Acidiferrobacteraceae e.g., *Sulfurifustis*
B	1.1	Long	Yes	Long	Yes	Fused with ctaB, Cys pair 2 separated by 8-9 aa	*Bdellovibrio* & *Halobacteriovorax*, alphaproteobacteria MAG, other Proteobacteria
B	1.2	Long	Yes	Very short	Yes	Substitution H278D *	Aquificae e.g., *Thermocrinis*
C	1.3	Medium	Yes	Short	No	Substitution H60N *	Actinobacteria: Acidimicrobiaceae; Proteobacteria MAG
A	1.4	Medium	Yes			4TM, Cys pair separated by 6 aa	Archaea: TACK (*Aeropyrum pernix*), Euryarchaeota & Asgard
D	1.5	Medium to long	No	Long	No	Long ICL2	Verrucomicrobia, Acidobacteria, Chlorobi, *Ca.* Rokubacteria, *Ca.* Poribacteria, Omnitriophica, *Ca.* Division NC10, *Salinibacter*, *Ca.* Entotheonella, Planctomycetes
D	2.0	Extremely long	No	Long	No	Long ICL2	Gemmatimonadetes, Flavobacteria, *Ca.* Caldichraeota, Chloroflexi, Proteobacteria, mitochondria (Cox15)

The new classification of CtaA proteins here presented is expanded compared to that of reference (He et al., 2016) to include all the different types and subtypes that have been found in current versions of the NCBI database. The first column shows the alternative classification in classes A to D (Lewin and Hederstedt, 2016). ECL1 and ECL3 indicate major extracellular loops while ICL2 indicates intracellular loop 2. See Figure 2 for structural models of various types of CtaA. ∼ (Niwa et al., 2018), ^ (Zeng et al., 2020), * (B. subtilis CtaA residue number; see Figure 2A).

**FIGURE 3:**
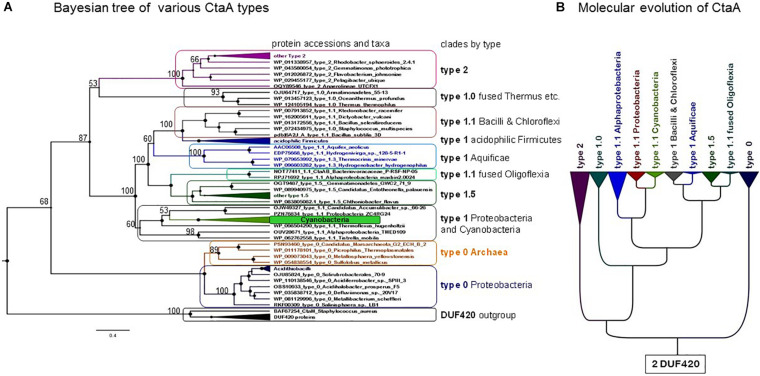
Phylogeny and evolution of heme A synthase. **(A)** Bayesian Maximal Clade Credibility (MCC) tree obtained with the BEAST package using an alignment of 53 CtaA and 7 DUF420 protein sequences as the outgroup. A similar topology was seen in ML trees obtained with the same alignment and different methods (Supplementary Figure 2 and results not shown). The alignment included 358 amino acid positions. Posterior support is expressed by the size of the dark circles on the nodes, the largest size representing 1 = 100%. Percentage values adjacent to various nodes are additionally shown. **(B)** Scheme for the molecular evolution of CtaA modelled on the silhouette of the Bayesian tree of bacterial CtaA proteins shown in Supplementary Figure 3. A primordial gene (presumed to encode a 4TM DUF420 protein) was duplicated and fused in tandem to give rise to a gene encoding a type 0-like CtaA protein. During subsequent evolution of CtaA, pairs of cysteine residues in ECL1 and ECL3 have been acquired and lost, depending on the lineage.

Our comprehensive new classification of heme A synthases (Table 1) is based upon the integration of the molecular features found in all variants present in current NCBI protein resources, including recently added metagenome data, and the consistent phylogenetic pattern of the variants that emerged from multiple approaches of phylogenetic analysis (detailed in the Supplementary Material). The Bayesian tree shown in Figure 3A represents a condensed view of the current phylogenetic distribution of CtaA proteins in prokaryotes, encompassing diverse representatives not only of type 1 and 2, but also members of two variants that we defined recently (Degli Esposti et al., 2020). One such variant is a derivative of type 1, called type 1.5. The other variant could not be fitted in either type 1 or type 2 and hence is called type 0 (Table 1). The type 0 CtaA proteins are very divergent in sequence but contain the invariant residues that are considered crucial for activity of heme A synthases (Hederstedt, 2012) (Figure 2B) and their genes are often present at the end of either the *rus* operon encoding COX subunits (Figure 1C) or the cytochrome *bo*_3_ operon of acidophilic Fe^2+^-oxidizers (Appia-Ayme et al., 1999; Quatrini et al., 2009; Issotta et al., 2018).

**FIGURE 4:**
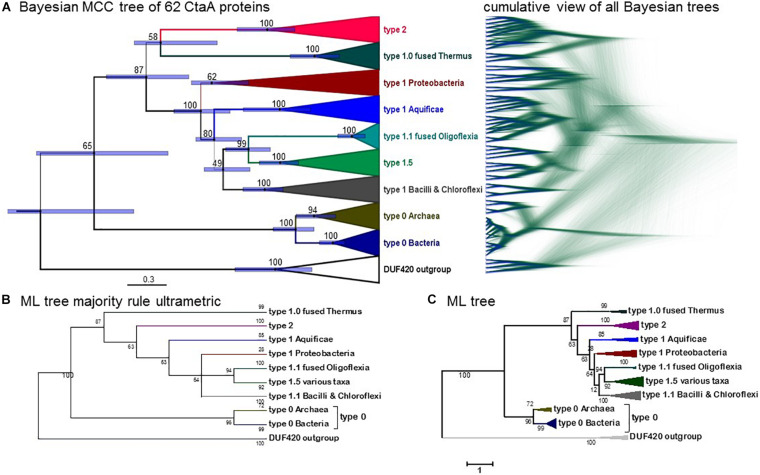
Phylogeny of CtaA proteins from bacterial and archaeal taxa. **(A)** Bayesian MCC tree obtained from an alignment of 58 CtaA and 4 DUF420 sequences as in Figure 3A (for a total of 358 amino acid sites), with a chain length of 6.2 million iterations; left, BEAST MCC tree with posterior support in% and node bars expressing the 95%_HPD posterior probability, equivalent to confidence intervals (Lee et al., 2013); right, overlaid 5581 trees in mirror image with the DensiTree program. **(B)** Majority rule-like ML tree obtained with the same CtaA alignment as in Panel **(A)** using the MEGA5 program (1000 bootstraps) by collapsing clades with less than 50% support. **(C)** ML tree obtained with MEGA5 as in Panel **(B)** without further manipulation. Similar ML trees were obtained with other programs such as IQ-Tree.

Type 0 CtaA consistently forms the basal branch in phylogenetic trees comprising all types of CtaA proteins (Figures 3A, 4 and Supplementary Figures 2–5). This pattern suggests a scheme for the molecular evolution of CtaA proteins (Figure 3B), which fundamentally follows the branching order of Bayesian (ultrametric) trees obtained from comprehensive alignments of the bacterial variants of CtaA. We hypothesize that the gene for a four TM DUF420 protein might have been duplicated and fused to form the gene for the ancestor of all current CtaA proteins (Figure 3B). Type 0 CtaA may constitute the extant descendant of such an ancestral protein, from which the type 2 and type 1 CtaA branched off. This branching might have occurred simultaneously, forming the sister clades that are frequently observed in phylogenetic trees (Figures 3, 4 and Supplementary Figure 3; He et al., 2016; Degli Esposti et al., 2020), or in a rapid sequence of differentiation, in which the cysteine pairs of current type 1.1 CtaA might have been acquired gradually, as suggested by some Maximum Likelihood (ML) trees (Supplementary Figure 4). The latter possibility is sustained by the occurrence of type 1.0 CtaA proteins having only the first cysteine pair and a long ECL1 comparable to that of type 2 CtaA (Table 1). These proteins are clearly different from type 1.0 proteins such as that of *S. aureus*, which most likely derive from a recent loss of the cysteine pair in ECL3, since they retain the same extracellular loops and closely cluster with type 1.1 CtaA proteins of related taxa such as *B. subtilis* (Table 1, Figures 3, 4 and Supplementary Figures 3–5). Similarly, type 1.5 CtaA likely derives from secondary losses of both cysteine pairs from type 1.1 proteins of Proteobacteria. As known from experiments with *B. subtilis* CtaA, the two cysteine residues in ECL1, but not those in ECL3, are important for heme A synthase activity (Lewin and Hederstedt, 2016) and the size of ECL3 can be changed without loss of enzyme activity (Lewin and Hederstedt, 2008).

In summary, our detailed analysis [see Supplementary Material for the evaluation of potential problems arising from Long Branch Attraction, LBA (Brinkmann et al., 2005; Philippe et al., 2005; Bleidorn, 2017)] strongly suggests that type 0 CtaA constitutes the ancestral form of heme A synthase, or a relative thereof (Figure 3B). Next, we explored the current taxonomic distribution of these proteins that, following our rationale, would define extant taxa possessing early divergent forms of COX.

### Taxonomic Distribution of Ancestral CtaA and Ecological Niches for Bacteria With This Type of CtaA

Blast searches with type 0 CtaA protein sequences against the entire nr database produced significant hits with closely related proteins and a few outgroup proteins. Currently there are about 100 recognized type 0 CtaA proteins, the majority of which is coded by the genomes of prokaryotes that share niches of acid soil or hydrothermal environments (Quatrini and Johnson, 2018; Supplementary Figure 6). The taxa of Proteobacteria with acidophilic iron- and sulfur-oxidizing physiology (*Acidithiobacillus*, *Acidiferrobacter* and *Acidihalobacter* spp.) contain the gene for type 0 CtaA as the sole heme A synthase. Intriguingly, several *Acidithiobacillus* species that lack the *rus* operon with COX genes also have type 0 CtaA orthologs (Acuña et al., 2013; Issotta et al., 2018). In these taxa, which include *At. caldus*, *At. sulfuriphilus* and *At. thiooxidans*, the gene for type 0 CtaA is usually found at the end of operons encoding orthologs of cytochrome *bo*_3_ ubiquinol oxidases. Identification of cytochrome *a* in the membranes of *At. thiooxidans* and the isolation of a cytochrome *aa*_3_ ubiquinol oxidase from the same bacterium (Sugio et al., 2006) suggest that these *Acidithiobacillus* spp. have heme A-containing quinol oxidases similar to *B. subtilis* cytochrome *aa*_3_. The distribution of genes for type 0 CtaA is scattered on the chromosome of other Proteobacteria that are not strongly acidophilic.

Biochemical studies on COX have been reported for several thermoacidophilic archaea that often share the same acidic environments with *Acidithiobacillus* spp., i.e., *Sulfolobus* (Lübben and Morand, 1994; Castresana and Saraste, 1995; Yarzábal et al., 2004) and *Ferroplasma* (Yarzábal et al., 2004; Castelle et al., 2015; Blake et al., 2016). However, biochemical information is not currently available for the other prokaryotes that contain type 0 CtaA. Consequently, we had to apply deductive approaches of genomic and sequence analysis to rationalize the peculiar taxonomic distribution of type 0 CtaA and understand its possible relationship with the evolution of COX.

### Archaea May Be Excluded From the Ancestry of Heme A-Containing Oxidases

Given the common ecophysiology of the organisms having type 0 CtaA, two alternative hypotheses can explain their current distribution (Supplementary Figure 6). The first hypothesis is that archaea first evolved CtaA and consequently heme A-containing oxidases and then passed the genes to bacteria. The second hypothesis is that ancestors of extant acidophilic bacteria evolved CtaA and COX first and their genes were transferred by LGT to archaeal lineages sharing their same environment. We examined the first hypothesis thoroughly, considering recent reports of Thermoplasmatales oxidases forming the basal branch in COX1 trees (Golyshina et al., 2016; Spang et al., 2019), which would strengthen the idea of archaeal ancestry for COX (Hemp and Gennis, 2008; Ducluzeau et al., 2014). Our phylogenetic analysis focused on archaeal COX proteins of family A. The COX1 proteins of the *phylum Ca.* Marsarchaeota (Jay et al., 2018) are fusion proteins with a highly divergent form of COX3 and contain two extra TM at the N-terminal end (Supplementary Table 1). This is an ancestral feature shared with diverse bacterial COX1 sequences (Degli Esposti, 2020), but not seen in other archaeal COX1 proteins such as SoxM, which also are COX1-3 fusion proteins (Pereira et al., 2001; Komorowski et al., 2002). Conversely, COX proteins of Cuniculiplasmataceae have been considered to be ancestral to the whole family A (Golyshina et al., 2016). We confirmed the basal position of these variants and found an extra TM at their N terminal end, but noted that genomes of Cuniculiplasmataceae spp. do not contain a gene for CtaA. Hence, their COX likely represents an intermediate in the transition between family A and B oxidases.

**FIGURE 5:**
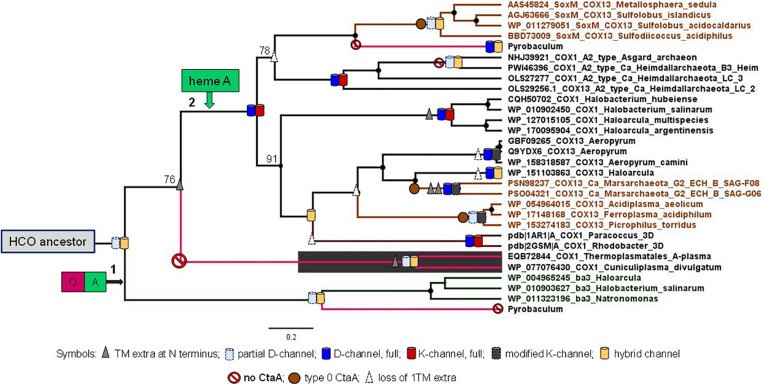
Phylogeny of archaeal COX1. Bayesian MCC tree obtained with the BEAST package using an alignment of 28 archaeal COX1 sequences of both the family A and B and two bacterial COX1 from Rhodobacteraceae with known 3D structure as described in the Supplementary Material. The final alignment included 558 amino acid sites. Posterior branch support was 100% except for the three cases where numerals are shown adjacent to specific nodes. The distribution of an extra TM at the N terminal end and of the different variants of the K-channel classified in Supplementary Figure 7, as well as of the D-channel (Degli Esposti et al., 2020), were annotated onto the tree branches using the symbols shown at the bottom of the figure.

Once mapped upon a robust phylogenetic tree of family A and B oxidases (Figure 5), the distribution of an extra TM at the N terminal end and the variants of both the K- and D-channels for proton pumping (Supplementary Figure 7) indicate that the ancestor of archaeal heme A-containing oxidases diverged after the separation of Cuniculiplasmataceae from the ancestral lineage connecting to family B (node 2 in Figure 5). If this hypothetical proto-archaeal lineage contained the ancestor of all heme A-containing oxidases, then the feature of an extra TM at the N terminal end would have been lost at least five times along their evolution. The same ancestor must have possessed the proton conducting K-channel, because the canonical form of this channel is present in both major lineages departing from its node (Figure 5 and Supplementary Figure 7). Consequently, the K-channel would have been independently lost seven times along the evolution of family A oxidases (Figure 5). Moreover, the presence of type 0 CtaA in organisms belonging to both major clades of archaeal family A oxidases implies multiple losses, if its gene were present in the archaeal ancestor (Figure 5). The appraisal of the predictions and implications applied to the tree and data in Figure 5 indicates that the second hypothesis, namely that current archaeal genes for type 0 CtaA proteins have been acquired from bacteria via multiple LGT events, is much more plausible than the first hypothesis stating the opposite. Indeed, several works have suggested or reported LGT events from bacteria to archaea, especially for proteins associated with bioenergetics such as COX (Boucher et al., 2003; Blank, 2009, 2019; Nelson-Sathi et al., 2012, 2015; López-García et al., 2015; Wolfe and Fournier, 2018). Together with our integrated analysis (Figure 5), this evidence for LGT essentially excludes the first hypothesis.

**FIGURE 6:**
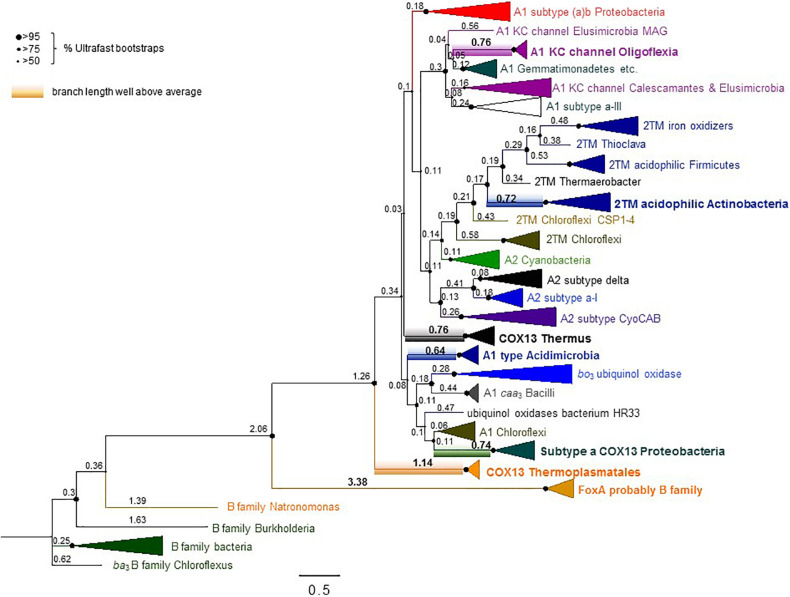
Extended phylogenic tree of COX1 from many bacteria and Thermoplasmatales archaea. The ML tree was obtained with the program IQ-Tree using an alignment of 100 COX1 sequences from family A and B oxidases encompassing most subclades analyzed here (Supplementary Table 1). The alignment had 607 amino acid sites and was analyzed with the LG model. Similar trees were obtained with a slightly more trimmed version of the same alignment (Supplementary Figure 8a and separate supplementary file COX1refined100IQTreeEX_EHOFigTreefull.pdf), which had stronger values of%Ultrafast bootstraps support (indicated here by the size of the nodes shown on the left of the figure). The focus of this figure is to show the values for the relative branch length of the various subclades, annotated as raw values obtained from the FigTree program. See Supplementary Figure 8b for the statistical analysis of these values. Note the large values of branch length (highlighted in bold numerals) for the clades including the proteins from Thermoplasmatales and similarly COX1-3 fusion proteins from Proteobacteria and *Thermus* spp.

Although Thermoplasmatales proteins appear to occupy the most basal branch of COX1 in phylogenetic trees (Figure 6, see also Supplementary File COX1refined100IQTreeEX_EHOFigTreefull.pdf), their clade exhibits a very long branch (Figure 6 and Supplementary Figure 8). An equivalent long branch was observed in ML trees obtained with various methods and tended to cluster together with long branches of bacterial COX1-3 fusion proteins such as those of *Thermus* spp. (Figure 6, cf. Supplementary Figure 8). As mentioned earlier in connection with CtaA, attraction between protein clades with long branches is a recurrent artifact in phylogenetic analysis (Brinkmann et al., 2005; Philippe et al., 2005; Bleidorn, 2017). Our analysis suggests that the basal position of Thermoplasmatales COX1-3 proteins in trees encompassing most COX subclades analyzed here (Figure 6), as well as in previously published phylogenetic trees (Spang et al., 2019), likely derives from LBA artifacts. Such problems probably arise from the different evolutionary rate of archaeal and bacterial proteins (Blank, 2009, 2019; Da Cunha et al., 2017; Cavalier-Smith and Chao, 2020). Moreover, the basal position of Thermoplasmatales COX1 in phylogenetic trees (Figure 6 and Supplementary Figure 8a) does not reflect their phylogenetic position in species trees of archaea (Blank, 2009; Colman et al., 2018; Wolfe and Fournier, 2018), which is consistent with the relatively recent age of these thermoacidophilic prokaryotes (Blank, 2009; Colman et al., 2018).

### Toward a Resolution of the Phylogeny of Bacterial COX1

Given that archaea can be excluded from the ancestry of COX, we next focused our work on bacterial COX to resolve its phylogeny, following the reductionist approaches presented in the Supplementary Material. We started from the observation that no bacterial genome could be found without the concomitant presence of CtaA and family A COX1 genes. We have analyzed all forms of heme A-containing oxidases in currently available genome sequences (Supplementary Table 1) but focus here on those belonging to family A for several reasons. This family is by far the most widespread of the HCO superfamily among living organisms and it encompasses the majority of prokaryotic lineages (see Degli Esposti, 2020 and references therein). In several bacterial taxa, the gene for heme A synthase is associated with the gene cluster of the COX subunits of family A HCO (Degli Esposti, 2020), while this is rarely the case for family B oxidases (Degli Esposti et al., 2019). Furthermore, prokaryotic genomes that encode a single terminal oxidase related to family B, as members of the Cuniculiplasmataceae, do not possess a gene for heme A synthase (Figure 5). Finally, and most importantly, family B oxidases do not present the ancestral features of an extra TM at the N-terminal end and residues typical of the K^*C*^-channel that are shared by family A and C oxidases (Degli Esposti, 2020 – see also Supplementary Figure 7).

**FIGURE 7:**
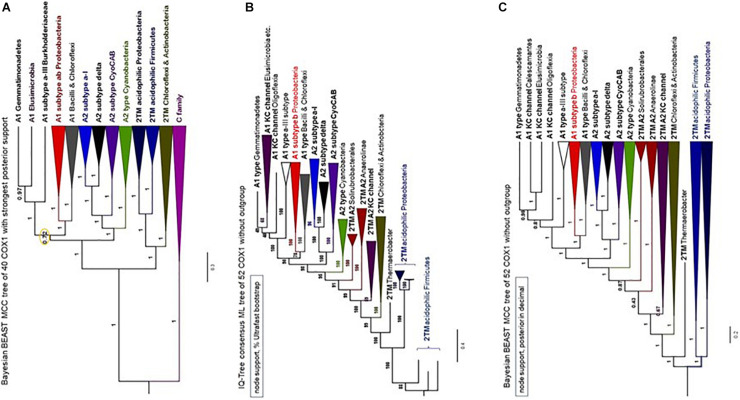
Phylogeny of COX1 with and without family C outgroup. The illustration combines phylogenetic trees of COX1 obtained with similar alignments of COX1 sequences for a total of 647 amino acid sites. **(A)** MCC tree obtained with the BEAST program using a chain of 2 million iterations and the BLOSUM62 model applied to an alignment of 37 COX1 and three FixN protein sequences that was refined with the new approach presented in the Supplementary Material (in particular, Supplementary Figures 11, 12). The node indicated with the orange circle is the only one with posterior support below 0.97 (i.e., less than 97%) and was implemented from the tree shown in the related Supplementary Figure 12a. **(B,C)** Phylogenetic trees obtained with the IQ-Tree program in Panel B and the BEAST program in Panel **(C)** using the same WAG model as in Panel **(A)** and an alignment of 52 COX1 sequences without FixN paralogs as outgroup. The programs and node support systems are presented on the top left of each panel. Additional trees obtained with other programs and the same alignment (available upon request) are shown in Supplementary Figures 12a,c. The Bayesian tree in Panel **(C)** was re-rooted to the branch of acidophilic iron-oxidizers.

The phylogeny of bacterial COX1 is much more complicated than that of archaeal COX1, fundamentally because family A oxidases have a very broad phylogenetic distribution encompassing diverse bacterial lineages (Degli Esposti, 2020; Pereira et al., 2001). We have analyzed all COX1 variants found in available genome sequences and found that the great majority of the variants can be divided in about twenty bacterial subclades (Supplementary Table 1). Split gene clusters typically found in late diverging Actinobacteria, such as *Corynebacterium* spp., have been previously found to cluster with one of the subclades described in Supplementary Table 1 (Degli Esposti, 2020), and were not analyzed here. After this initial selection, we progressively removed the subclades with long branches or fast substitution rate, that were present in phylogenetically broad trees such as that in Figure 6. This strategy was integrated with a novel approach to build COX1 alignments encompassing the N-terminal region so as to minimize LBA artifacts in assessing the phylogeny of bacterial COX1, as described in the dedicated section of Supplementary Material. Using multiple methods of phylogenetic inference, we ultimately concluded that the Bayesian tree shown in Figure 7A represents a most robust phylogeny of bacterial COX1. The tree presents an early separation of the A1 and the A2 type of COX1 from the basal clade containing COXI with 2 additional TM at the N-terminal end (these proteins are abbreviated 2TM) of various lineages, including iron-oxidizing bacteria. This phylogeny was confirmed in ML trees obtained with the same refined alignment (Supplementary Figures 9–12). Moreover, an equivalent topology emerged in phylogenetic trees obtained without family C paralogs as outgroup (Figures 7B,C). Hence, the choice of outgroup proteins used in previous phylogenetic trees does not affect the consistent basal position of the proteins with the ancestral 2TM extra feature, which include all those of iron-oxidizers that have type 0 CtaA.

### Acidophilic Iron-Oxidizers Have an Ancestral Form of the COX Assembly Protein CtaG

**FIGURE 8:**
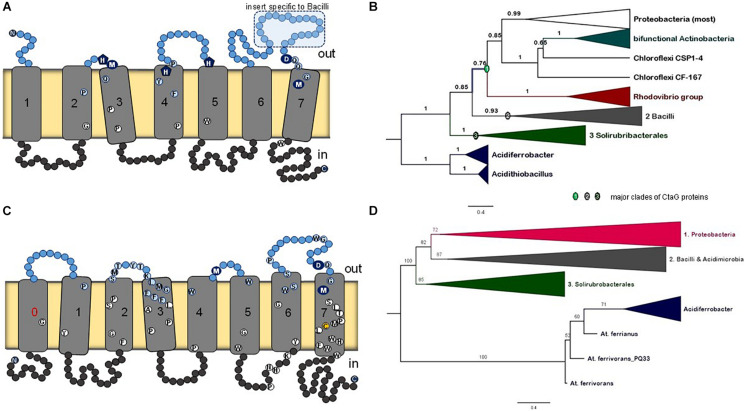
Membrane structure and phylogeny of caa3_CtaG proteins. **(A)** Schematic model for the deduced transmembrane structure of *B. subtilis* caa3_CtaG, which was rendered with the same design as that used for CtaA (Figure 2). Residues potentially acting as Cu ion ligands are indicated with blue circles or pentagons including the invariant D249 (see text). Other highly conserved residues in caa3_CtaG proteins are depicted as in Figure 2. **(B)** Representative Bayesian tree obtained with the BEAST package using 40 caa3_CtaG sequences that were selected to represent the major clades previously found in wide phylogenetic analysis after removing proteins exhibiting long branches (Supplementary Figures 13, 14a). A similar tree was obtained with ML inference using the IQ-Tree program (Supplementary Figure 14b). The alignment contained a total of 242 amino acid sites. Posterior support of branches is expressed in decimals. **(C)** Schematic model for the structure of caa3_ctaG of acidophilic iron-oxidizing Proteobacteria, exemplified by that of *Acidithiobacillus ferrianus* (Norris et al., 2020). **(D)** The ML tree was obtained with the IQ-Tree program using an alignment of 42 caa3-CtaG sequences including their N terminal part [i.e., not trimmed as in Panel **(B)**]. The alignment contained a total of 337 amino acid residues and is made available upon request.

CtaG is required for the assembly of Cu_B_ in COX1 (Figure 1A). It must be noted that there are two different types of proteins in bacteria that are called CtaG. One is a homolog of eukaryotic Cox11 and present in, for example, *P. denitrificans*. We present an analysis of this type of protein and its phylogenetic distribution in a dedicated section of the Supplementary Material. The other type is named caa3_CtaG and present in, for example, *B. subtilis* (Bengtsson et al., 2004). The caa3_CtaG constitutes the focus of our analysis for several reasons. It is a membrane protein with multiple TM (Figure 8A) and its function is intertwined with that of CtaA to form the heme *a*_3_-Cu_B_ oxygen-reacting center in COX1. Moreover, the gene for caa3_CtaG often clusters with genes for COX in the chromosome (Figure 1C) of taxa that have the deep branching form of COX1 with the 2TM extra feature (Supplementary Tables 1, 2). Considering all these points, the molecular evolution of caa3_CtaG is likely connected with the early evolution of COX in bacteria, while that of Cox11 homologs may be more relevant to the bacterial ancestry of mitochondrial proteins (Degli Esposti et al., 2019).

There is no available structure for caa3_CtaG nor any paralog. The taxonomic distribution of caa3_CtaG is narrower than that of CtaA. To discern the molecular evolution of caa3_CtaG proteins, we undertook a systematic phylogenetic analysis of all the proteins that show the caa3_CtaG domain and have 6 to 9 TM (Supplementary Table 2). The most common structure is similar to that of *B. subtilis* caa3_CtaG, with 7 TM (Figure 8A). There are several conserved amino acid residues at the predicted positive (outer side) side of the membrane and which might function as Cu ligands (Degli Esposti et al., 2020), among which aspartate residue 249 appears to be invariant. The number of TM does not necessarily correlate with the phylogenetic position of caa3_CtaG proteins, which consistently cluster in three major clades (Figure 8B and Supplementary Figures 13, 14).

The possible phylogeny of caa3_CtaG proteins is shown by the tree of Figure 8B in its simplest and most robust form. This tree was reconstructed from an alignment of the sequences of 40 diverse proteins that represent the taxonomic breadth of the major clades of caa3_CtaG (Supplementary Table 2) while minimizing the presence of long branches, as described in the dedicated section of the Supplementary Material. The caa3_CtaG proteins of *Acidithiobacillus* spp. and *Acidiferrobacter* spp., which are predicted to have 8TM (one additional TM at the N-terminal end), show a different set of potential copper ion ligands than those in most other caa3_CtaG proteins (Figure 8C). However, these proteins maintain the invariant aspartate 249 (*B. subtilis* caa3_CtaG numbering) at the positive side of the membrane and are recognized as members of the caa3_CtaG superfamily. Using alignments that included also the N-terminal part of caa3_CtaG proteins, which has been routinely trimmed before (as in the alignment used to generate the tree in Figure 8B, cf. [Degli Esposti et al., 2020]), we obtained similar phylogenetic trees (Figure 8D and Supplementary Figure 14). Hence, the basal position of caa3_CtaG of iron-oxidizing Proteobacteria such as *Acidithiobacillus* spp. was confirmed in multiple ways, consistent with the molecular evolution of CtaA (Figure 3B).

## Conclusion

This work presents converging evidence suggesting that COX of extant acidophilic bacteria, in particular iron-oxidizing Proteobacteria such *Acidithiobacillus* and *Acidiferrobacter* spp., may be the closest to primitive heme A-containing respiratory oxidases. Protein factors specifically involved in biosynthesis of COX have presumably coevolved with COX1, as reflected by the clustering of their genes with those encoding COX proteins. The results of our in depth analysis of two proteins required for the assembly of the oxygen-reacting center in COX, CtaA for the biogenesis of heme A and caa3_CtaG for Cu_B_, strongly support the inferred phylogeny of bacterial COX. Namely, CtaA and caa3_CtaG proteins of iron-oxidizing Proteobacteria form the basal branch in phylogenetic trees obtained with different inference methods and programs of phylogenetic analysis. Once invented, the advantageous bioenergetic capacity of respiration with molecular oxygen via heme A-containing oxidases evidently spread to various prokaryotes (Han et al., 2011; Wikström and Springett, 2020).

Geochemical and ecological evidence sustains the COX phylogeny that emerges from our data. Lithotrophic bacteria, such as *Acidithiobacillus* spp. and other acidophilic iron-oxidizers can release abundant levels of Cu ions by bioleaching of common crust rocks (Quatrini and Johnson, 2018; Degli Esposti et al., 2020). Cu bioavailability often limits COX biogenesis in aquatic environments, especially in oceans where Cu ion concentrations are normally low (Degli Esposti et al., 2019). The earliest geochemical evidence for bacterial respiration points to ancestral iron-oxidizing bacteria similar to extant *Acidithiobacillus* spp. as responsible for the acid leaching of soil crust rocks containing metals such as Cu, Cr and Co, which then were washed away from emerged land producing rich deposits in ocean sediments (Konhauser et al., 2011). Metal leaching driven by bacterial respiration lasted only a few hundred million years during and after the GOE (Konhauser et al., 2011). Once surface pyrite minerals were consumed by intense oxidation, COX genes had spread laterally to soil dwelling bacteria with faster growth capacity than the ancestors of extant iron oxidizers. To conclude, the early evolution of COX was apparently promoted by the availability of relatively high levels of oxygen produced locally by Cyanobacteria, combined with the availability of surface pyrite rock material in primordial earth environments.

## Data Availability Statement

The original contributions presented in the study are included in the article/Supplementary Material; further inquiries can be directed to the corresponding author/s.

## Author Contributions

MDE had the original idea for the study and initiated the writing of the manuscript, progressively involving the other co-authors who are experts in specific areas that are encompassed by this multi-disciplinary manuscript, structured most of the figures and tables. AM-B performed various phylogenetic analysis of COX assembly proteins. RQ is an expert on the family of Acidithiobacillaceae and contributed genomic and taxonomic data and did bioinformatic analyses. LH contributed expertise on heme A synthase and molecular microbiology information to frame and structure the manuscript. LH and MDE co-wrote the final manuscript. All authors contributed to the article and approved the submitted version.

## Conflict of Interest

The authors declare that the research was conducted in the absence of any commercial or financial relationships that could be construed as a potential conflict of interest.

## Footnotes

1 https://www.ncbi.nlm.nih.gov/Structure/cdd/

2 http://tree.bio.ed.ac.uk/software/figtree/

3 http://www.sacs.ucsf.edu/TOPO2/
