# Supplementary material for: Respiratory Heme A-Containing Oxidases Originated in the Ancestors of Iron-Oxidizing Bacteria
Source: Front Microbiol. 2021 Jun 15;12:664216. doi: 10.3389/fmicb.2021.664216 (PMC8239418; doi:10.3389/fmicb.2021.664216)
Supplement: Supplementary file 1 [file Data_Sheet_1.PDF]

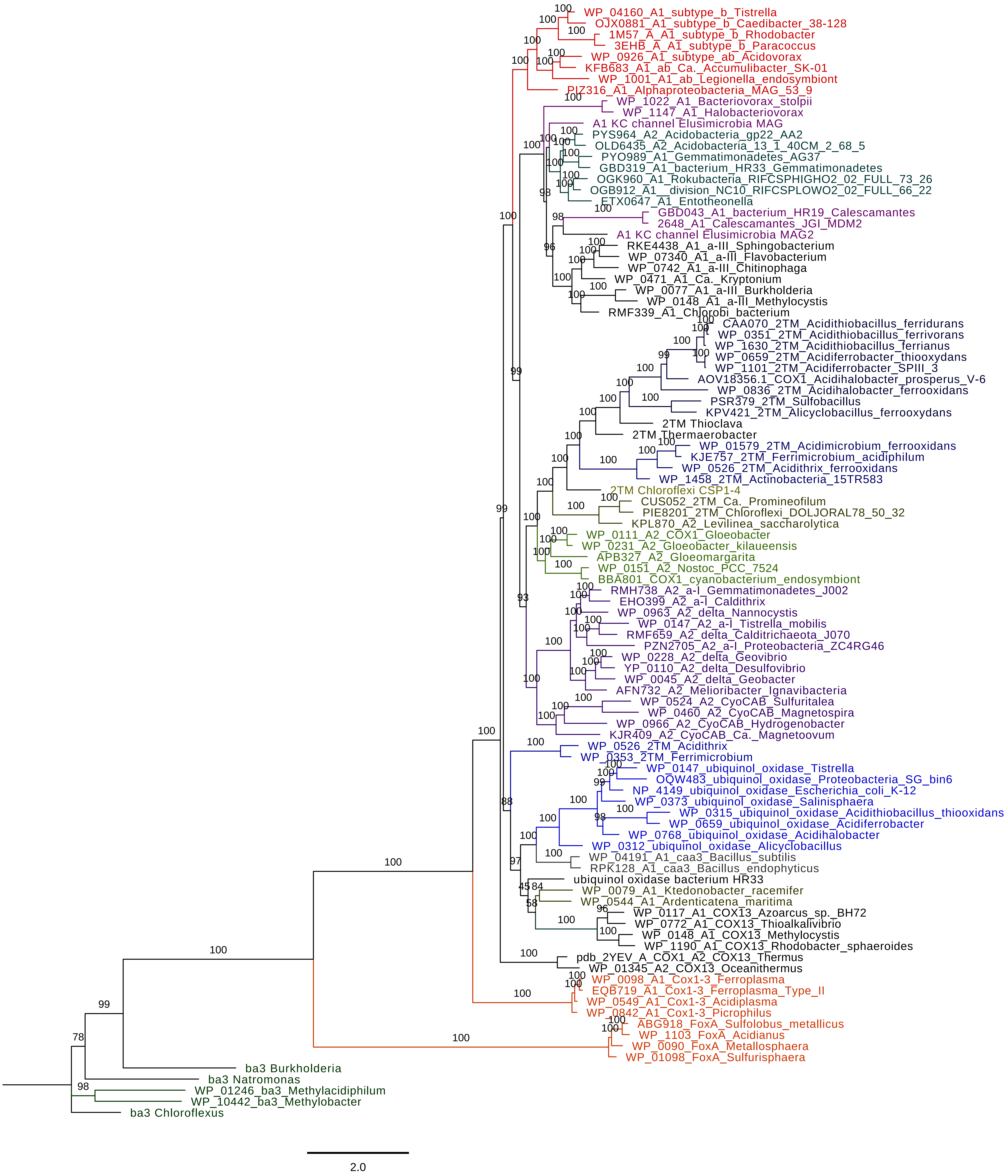

WP_04160_A1_subtype_b_Tistrella
OJX0881_A1_subtype_b_Caedibacter_38-128
1M57_A_A1_subtype_b_Rhodobacter
3EHB_A_A1_subtype_b_Paracoccus
WP_0926_A1_subtype_ab_Acidovorax
KFB683_A1_ab_Ca._Accumulibacter_SK-01
WP_1001_A1_ab_Legionella_endosymbiont
PIZ316_A1_Alphaproteobacteria_MAG_53_9
WP_1022_A1_Bacteriovorax_stolpii
WP_1147_A1_Halobacteriovorax
A1 KC channel Elusimicrobia MAG
PYS964_A2_Acidobacteria_gp22_AA2
OLD6435_A2_Acidobacteria_13_1_40CM_2_68_5
PYO989_A1_Gemmatimonadetes_AG37
GBD319_A1_bacterium_HR33_Gemmatimonadetes
OGK960_A1_Rokubacteria_RIFCSPHIGHO2_02_FULL_73_26
OGB912_A1__division_NC10_RIFCSPLOWO2_02_FULL_66_22
ETX0647_A1_Entotheonella
GBD043_A1_bacterium_HR19_Calescamantes
2648_A1_Calescamantes_JGI_MDM2
A1 KC channel Elusimicrobia MAG2
RKE4438_A1_a-III_Sphingobacterium
WP_07340_A1_a-III_Flavobacterium
WP_0742_A1_a-III_Chitinophaga
WP_0471_A1_Ca._Kryptonium
WP_0077_A1_a-III_Burkholderia
WP_0148_A1_a-III_Methylocystis
RMF339_A1_Chlorobi_bacterium
CAA070_2TM_Acidithiobacillus_ferridurans
WP_0351_2TM_Acidithiobacillus_ferrivorans
WP_1630_2TM_Acidithiobacillus_ferrianus
WP_0659_2TM_Acidiferrobacter_thiooxydans
WP_1101_2TM_Acidiferrobacter_SPIII_3
AOV18356.1_COX1_Acidihalobacter_prosperus_V-6
WP_0836_2TM_Acidihalobacter_ferrooxidans
PSR379_2TM_Sulfobacillus
KPV421_2TM_Alicyclobacillus_ferrooxydans
2TM Thioclava
2TM Thermaerobacter
WP_01579_2TM_Acidimicrobium_ferrooxidans
KJE757_2TM_Ferrimicrobium_acidiphilum
WP_0526_2TM_Acidithrix_ferrooxidans
WP_1458_2TM_Actinobacteria_15TR583
2TM Chloroflexi CSP1-4
CUS052_2TM_Ca._Promineofilum
PIE8201_2TM_Chloroflexi_DOLJORAL78_50_32
KPL870_A2_Levilinea_saccharolytica
WP_0111_A2_COX1_Gloeobacter
WP_0231_A2_Gloeobacter_kilaueensis
APB327_A2_Gloeomargarita
WP_0151_A2_Nostoc_PCC_7524
BBA801_COX1_cyanobacterium_endosymbiont
RMH738_A2_a-I_Gemmatimonadetes_J002
EHO399_A2_a-I_Caldithrix
WP_0963_A2_delta_Nannocystis
WP_0147_A2_a-I_Tistrella_mobilis
RMF659_A2_delta_Calditrichaeota_J070
PZN2705_A2_a-I_Proteobacteria_ZC4RG46
WP_0228_A2_delta_Geovibrio
YP_0110_A2_delta_Desulfovibrio
WP_0045_A2_delta_Geobacter
AFN732_A2_Melioribacter_Ignavibacteria
WP_0524_A2_CyoCAB_Sulfuritalea
WP_0460_A2_CyoCAB_Magnetospira
WP_0966_A2_CyoCAB_Hydrogenobacter
KJR409_A2_CyoCAB_Ca._Magnetoovum
WP_0526_2TM_Acidithrix
WP_0353_2TM_Ferrimicrobium
WP_0147_ubiquinol_oxidase_Tistrella
OQW483_ubiquinol_oxidase_Proteobacteria_SG_bin6
NP_4149_ubiquinol_oxidase_Escherichia_coli_K-12
WP_0373_ubiquinol_oxidase_Salinisphaera
WP_0315_ubiquinol_oxidase_Acidithiobacillus_thiooxidans
WP_0659_ubiquinol_oxidase_Acidiferrobacter
WP_0768_ubiquinol_oxidase_Acidihalobacter
WP_0312_ubiquinol_oxidase_Alicyclobacillus
WP_04191_A1_caa3_Bacillus_subtilis
RPK128_A1_caa3_Bacillus_endophyticus
ubiquinol oxidase bacterium HR33
WP_0079_A1_Ktedonobacter_racemifer
WP_0544_A1_Ardenticatena_maritima
WP_0117_A1_COX13_Azoarcus_sp._BH72
WP_0772_A1_COX13_Thioalkalivibrio
WP_0148_A1_COX13_Methylocystis
WP_1190_A1_COX13_Rhodobacter_sphaeroides
pdb_2YEV_A_COX1_A2_COX13_Thermus
WP_01345_A2_COX13_Oceanithermus
WP_0098_A1_Cox1-3_Ferroplasma
EQB719_A1_Cox1-3_Ferroplasma_Type_II
WP_0549_A1_Cox1-3_Acidiplasma
WP_0842_A1_Cox1-3_Picrophilus
ABG918_FoxA_Sulfolobus_metallicus
WP_1103_FoxA_Acidianus
WP_0090_FoxA_Metallosphaera
WP_01098_FoxA_Sulfurisphaera
ba3 Burkholderia
ba3 Natromonas
WP_01246_ba3_Methylacidiphilum
WP_10442_ba3_Methylobacter
ba3 Chloroflexus
2.0
