# Supplementary material for: Respiratory Heme A-Containing Oxidases Originated in the Ancestors of Iron-Oxidizing Bacteria
Source: Front Microbiol. 2021 Jun 15;12:664216. doi: 10.3389/fmicb.2021.664216 (PMC8239418; doi:10.3389/fmicb.2021.664216)

Supplementary Material

Respiratory heme A-containing oxidases originated in the ancestors of iron-oxidizing bacteria

Mauro Degli Esposti, Ana Moya-Beltrán, Raquel Quatrini and Lars Hederstedt

The Supplementary Material with two tables and 15 supplementary figures includes additional information and display material for documenting our analysis of COX, CtaA and CtaG proteins. It presents also statistical analysis of phylogenetic trees that complements parts of the Materials and Methods. Additional files complementing Supplementary Figs. 7 and 8 are uploaded separately, similarly to the .excel files for the two Supplementary tables.

Analysis of CtaA proteins

In the present work, we expanded genomic surveys to evaluate all proteins classified within the Cox15-CtaA super-family (https://www.ncbi.nlm.nih.gov/Structure/cdd/cddsrv.cgi , accessed on 2 November 2020). We have scanned also recently deposited metagenome-assembled genomes (MAGs) from marine and aquatic environments to find new variants of CtaA that are present in genomes containing COX operons encoding family A oxidases, which were not considered in our previous analyses (Degli Esposti et al, 2020). After building a manually curated reference alignment encompassing all major types of CtaA proteins, we added any new candidate sequence obtained from BLAST searches, usually with E-values below 10^{-3} – except for the most distant type 0 CtaA proteins that are not picked by conventional searches. Sequences that showed substitutions of more than one of the five invariant residues that are considered important for CtaA function, namely a glutamic acid (E57) and four histidine residues that may ligate hemes (Hederstedt, 2012; Niwa et al, 2018), were annotated (cf. Table 1) but then removed from the alignment used for reconstructing phylogenetic trees. Conversely, we maintained sequences showing a conservative substitution of only one of such residues in some alignments used to reconstruct phylogenetic trees, such as that in Supplementary Figure S3. However, the alignments that were refined for reconstructing the trees shown in Figures 3 and 4 contained only sequences exhibiting the mentioned five invariant residues.

In our analysis, we initially kept as a reference the first published phylogenetic tree of CtaA proteins (He et al, 2016), which showed the deep separation of type 1 and type 2 into two major clades without an apparent root, with the CtaA of *Microcystis* sp., a freshwater taxon of Cyanobacteria, apparently forming

the deepest branch of type 1. After examining all the genomes of Cyanobacteria in current versions of National Center for Biotechnology Information (NCBI) repositories, we found that CtaA proteins of the *Microcystis* genus lie in the middle of a clade including *ca*. 800 full length proteins of Cyanobacteria that are currently available, the deepest branching of which is *Gloeobacter* CtaA (Figure 3A and Supplementary Figure S2a), in agreement with the phylogeny of the *phylum* Cyanobacteria (Uyeda et al, 2006). Moreover, cyanobacterial CtaA sequences are nested within a large clade in sister position to the clade including type 1.1 CtaA of Proteobacteria (Figure 3A and Supplementary Figures S2-S4).

Type 0 protein sequences are so divergent from other types of CtaA that they were (and still are) not easily recognized as members of the COX15-CtaA super-family. They were originally discovered as the product of a gene present at the end of the *rus* operon of certain iron-oxidizers (Figure 1B), cf. (Appia-Ayme et al, 1999; Issotta et al, 2018; Quatrini etal, 2009). CtaA proteins of *Acidithiobacillus* spp., together with those of other proteobacterial acidophilic Fe^{2+}-oxidizers such as *Acidiferrobacter* and *Acidihalobacter* spp., possess the invariant histidine and glutamate residues that are involved in heme binding, but lack the long ECL1 at the positive side of the membrane that is characteristic of type 2 CtaA (Figure 2). Our new results obtained using various approaches of phylogenetic inference and different taxonomic sampling produced diverse highly curated alignments (see Materials and Methods for further details) and trees built from them that strengthened our previous evidence for an ancestral character of type 0 CtaA (Degli Esposti et al, 2020).

The phylogenetic sequence of various CtaA types presented in the scheme of Figure 3B might be confounded, at least in part, by tree aberrations derived from long branches or unequal substitution rates across taxa. To evaluate such potential problems arising from, or labelled as, LBA for Long Branch Attraction (Bleidorn, 2017; Brinkmann et al, 2005; Philippe et al, 2005), we systematically analyzed the substitution rate and length properties of each branch in trees obtained with information-rich Bayesian analysis (Supplementary Figures S1 and S5); we additionally used mixture models of amino acid substitution for ML trees (Le et al, 2008, 2010) (Figure 4A and Supplementary Figure S4).

Short CtaA proteins of 4TM with similarity to either half of, for example, *B. subtilis* CtaA are characteristically present in various lineages of archaea, for example in *Aeropyrum pernix* (Lewin and Hederstedt, 2006). Such proteins were omitted from further analysis because they segregated with different bacterial clades depending upon the taxonomic composition and method used for reconstructing phylogenetic trees (Supplementary Figure S1, cf. [Degli Esposti et al, 2020]). Moreover, these short CtaA

proteins, now classified as type 1.4 (Table 1), showed long branches in phylogenetic trees (Supplementary Figure S1c), thereby producing systematic errors in their phylogenetic placement. As mentioned in the main text, we found a group of long-branch CtaA including rare proteins from Candidate Phyla Radiation (CPR), such as *Ca.* Margulisbacteria or *Ca.* Fraserbacteria, which have been picked up in Blast searches such as that shown in Supplementary Figure S6. These proteins do not represent defined CtaA subclades, since their position tend to vary in phylogenetic trees and belong to MAGs lacking complete COX operons. Moreover, the representative full length protein from *Ca.* Margulisbacteria (accession MSR88539, from a lake metagenome) might not be functional, since it lacks the first conserved histidine ligand for heme binding.

Four clades of bacterial type 1 CtaA consistently showed long branches in phylogenetic trees obtained using different approaches. The CtaA of *Thermus* spp. is the N terminal part of a bifunctional protein that includes CtaB, which segregates with similar fused proteins from Armatimonadetes. These proteins have been ignored previously (He et al, 2016) and are classified as type 1.0 here because they lack the cysteine pair in ECL3 (Table 1). They constitute a long-branch subclade (Supplementary Figure S1c) that most frequently clusters with the clade of type 2 CtaA (Figure 3A and Supplementary Figures S2 and S5). A second group of CtaA fused with CtaB encompasses proteins from Oligoflexia (family Bacteriovoraceae) and unclassified Proteobacteria, cumulatively labelled here as 'type 1.1 fused Oligoflexia' (Figures 3 and 4). These CtaA proteins show a wider separation between the two Cys residues in ECL3 than in most type 1.1 proteins (Table 1). The third group of long-branch CtaA includes proteins from Ca. Margulisbacteria and *Ca.* Fraserbacteria mentioned earlier. Finally, type 2 proteins exhibits a branch length that is usually larger than average (Supplementary Figure S1). Due to this feature, it is possible that the recurrent clustering of type 2 CtaA with the clade of fused type 1.0 from *Thermus* derives from LBA artifacts or other aberrations of phylogenetic trees. We addressed this issue by using mixture substitution models (e.g., [Le et al, 2010]) and found that both the clustering of the *Thermus* clade with the type 2 clade and that of the clade of type 1.1 fused Oligoflexia with the type 1.5 clade were maintained with strong support (Figure 4 and Supplementary Figure S4). However, clades of fused CtaA proteins always exhibited long branches, irrespective of the substitution model used for reconstructing the phylogenetic trees (Figure 4 and data not shown).

Another approach that is traditionally used to reduce LBA artifacts is to remove the sequences that exhibit long branches, eventually substituting them with related sequences (either structurally or taxonomically) to limit the inevitable information loss (Bleidorn, 2017). Following this approach, we

found that the removal of a single CtaA sequence or group of CtaA sequences exhibiting long branches did not significantly alter the overall tree topology. However, their removal generally reduced the support and topological reproducibility of internal nodes, especially in trees including 100 or more sequences encompassing all the taxa that have recognized CtaA proteins (Supplementary Figures S4 and S5a; see also [Degli Esposti et al, 2020]). Such taxonomically wide trees include proteins that show a substitution rate that is significantly higher than average, as previously found for other membrane-bound redox proteins (Khadka et al, 2018). This was the case, in particular, for two type 1 proteins form the genus *Alicyclobacillus* (acidophilic Firmicutes) that clearly differed from other CtaA of the same taxon (Supplementary Figure S5a and data not shown). Overall, however, the substitution rate was relatively similar in other proteins or sub-branches of CtaA trees, with the notable exception of *Andalucia* Cox15 (Supplementary Figures S3 and S4), presumably due to the notoriously fast rate of mutation of mitochondrial DNA. In any case, the basal position of the clade containing type 0 CtaA proteins was consistently found in phylogenetic trees obtained with different approaches, either in the presence or absence of the DUF420 outgroup (Figures 3 and 4, Supplementary Figures S3 and S4).

In the genome of strongly acidophilic Proteobacteria, genes for type 0 CtaA were found to be linked to COX genes on the chromosome. In other Proteobacteria that are not strongly acidophilic, and have diverse physiology, genes for type 0 CtaA are scattered on the chromosome. These taxa include the iron-metabolizing *Metallibacterium*, the mildly acidophilic and heterotrophic *Acidimangrovimonas sediminis* of the Rhodobacterales order of Alphaproteobacteria (Ren et al, 2019), the nitrite-oxidizing *Nitrococcus mobilis* and members of the *Salinisphaera* genus that have extremophilic character (Supplementary Figure S6). One gene for type 0 CtaA is present in a Solirubrobacterales MAG. Solirubrobacterales are a deep branching lineage of Actinobacteria found in soil environments (Hu et al, 2019) and have COX proteins that show features intermediate between Cyanobacteria and ancestral lineages (Degli Esposti, 2020). Solirubrobacterales and other non-acidophilic bacteria mentioned above additionally have genes for type 1 CtaA. Conversely, three lineages of thermoacidophilic archaea possessing type 0 CtaA have only that gene for heme A synthase (Supplementary Figure S6): the whole family of Ferroplasmaceae within Thermoplasmatales (Euryarchaeota) and that of Sulfolobaceae in the Sulfolobales order of Crenarchaeota (part of the TACK group), plus MAGs of *Ca.* Marsarchaeota (of the TACK group [Jay et al, 2018]).

Integrated approaches to resolve COX1 phylogeny

The evidence that type 0 CtaA may represents an ancestral form of heme A synthases would imply that the taxa that have the gene for this protein may also have deep branching forms of the major subunits of the COX enzyme, following the logical principle of co-evolution. This principle formed part of our methodological approaches permeated of reductionism (Bridandt and Love, 2007) to simplify the complex biological problem of finding the possible origin of COX. Focusing on the phylogeny of the major subunit COX1, we started with the A1 type present in thermoacidophilic archaea that also have type 0 CtaA (Supplementary Figure S6). Initially, the selection of these proteins was made following BlastP searches of *Halobacterium* COX1 against the nr database and represented the major lineages of aerobic archaea having family A oxidases (Blank, 2009; Sousa et al, 2012). Complementary BlastP searches were undertaken with COX1 proteins from other archaea such as *Ca.* Marsarchaeota. A large clade of proteins from marine Euryarchaeota MAG was omitted after finding that it clustered very closely to the COX1 of Betaproteobacteria (in the tree of Figure 5 they would thus cluster with the Rhodobacteraceae proteins). The full sequences were aligned with only minimal trimming of the N terminal part (generally restricted to a few residues for the longest proteins), first with the MUSCLE algorithm within the MEGA program and then refined manually to match the known structure of *P. denitrificans* COX as described in Materials and Methods. Once the alignment of 30 full sequences of COX1 had been refined, we applied both Bayesian and ML inference to reconstruct robust phylogenetic trees, on which we then annotated the distribution of CtaA types and other features that appear to be ancestral in the molecular evolution of COX1 (Degli Esposti, 2020), as shown in Figure 5.

After establishing that archaea may be excluded from the ancestry of COX (see main text), we focused our work on the possible resolution of the phylogeny of bacterial COX1 of the A family. This task initially appeared to be next to impossible, given the complexity of the molecular evolution of COX subunits that emerged from multiple previous studies (Degli Esposti et al, 2019; Ducluzeau et al, 2014; Golyshina et al, 2016; Han et al, 2011; Hemp et al, 2008; Pereira et al, 2001; Spang et al, 2019). However, the task was simplified by the exclusion of archaeal COX proteins from the likely ancestry of the COX enzyme (Fig. 5). We also excluded B family oxidases from further analysis for the reasons presented in the main text. We applied diverse approaches of sequence analysis to complement phylogenetic studies, realizing that phylogenetic trees alone can hardly resolve COX evolutionary patterns over long times such as that spanning the GOE (Degli Esposti, 2020). Our analysis was extended to all currently available bacterial genomes and produced a wealth of information that is summarized in Supplementary Table S1. The information is subdivided among the *ca.* 30 subclades of prokaryotic

COX1 that have been consistently found using diverse approaches of phylogenetic inference (see Materials and Methods for details) and often coincide with recognized operons (Supplementary Table S1, cf. [Degli Esposti, 2020]). The definition of such subclades subsequently guided the analysis of the phylogenetic trees reconstructed with bacterial COX1, and also with bacterial COX2, providing a simple consistent labeling for the branches containing proteins from different lineages sharing similar molecular features.

Given the broad taxonomic distribution of COX among diverse bacterial lineages, the choice of taxonomic sampling has paramount importance for reconstructing trees of COX sequences. We tackled this issue by carrying out a systematic search of COX1 variants to define the subclades and evaluate their main properties of branch length and substitution rate in phylogenetic trees (Supplementary Table S1). On the basis of such information, we subsequently excluded COX1-3 fusion proteins exhibiting long branches (Fig. 6 and Supplementary Figure S8). Late diverging COX1 proteins belonging to split gene clusters such as that of *Mycobacterium* spp. were also excluded for equivalent reasons. Next, we excluded the clade of ubiquinol oxidases corresponding to the cytochrome bo_3 subtype of A1 type COX (Degli Esposti et al, 2019) because their COX1 protein displayed a substitution rate that was much higher than that of other family A oxidases (Supplementary Figure S9B). We progressively narrowed the taxonomic sampling to a set of COX1 proteins that exhibited close to average values of either branch length or branch substitution rate, which in principle would reduce the potential problems related to LBA. Moreover, we verified that it was not necessary to use many members of the same subclade to provide a consistent placement of its branch in phylogenetic trees; in many cases, two members with significantly divergent sequence could define such a phylogenetic position. We thus settled on sets of 70 to 80 COX1 protein sequences, including a handful of FixN paralogs as outgroup, to conduct a thorough examination of the reproducibility of COX1 trees.

A crucial portion of the information in Supplementary Table S1 is derived from a wide survey of the molecular variation in the residues that form the proton-conducting channels in family A oxidases (Supplementary Figure S7), and expand the data in a previous study on the topic (Degli Esposti, 2020). Bacterial family A oxidases contain two proton-conducting channels that are fundamental for efficient energy conservation coupled to the reduction of oxygen in the catalytic cycle (Han et al, 2011; Pereira et al, 2001; Sharma and Wikström, 2014; Iwata et al, 1995). While the D-channel is apparently present only in the family A, the K-channel is common to family A, B, and C of the HCO superfamily. In family C, this channel is contributed by two sets of protonable amino acid residues, one specific to the family and

one in common with the family A (Degli Esposti, 2020), and has been frequently labeled K^C-channel (Ahn et al, 2018). Moreover, protonable amino acid residues are present in equivalent positions of those specific to the K^C-channel in several COX1 proteins of family A oxidases, but not B family oxidases, in a pattern supporting the hypothesis that these oxidases gradually evolved from family C oxidases (Castresana and Saraste, 1995). See reference (Degli Esposti, 2020) for a recent review.

The amino acid residues of the K-channel reflect the functional evolution of COX without clear instances of co-evolution in residues that are spatially adjacent to each other (Wang and Pollock, 2007). Such residues are often located in different TM of the protein (Ahn et al, 2018; Iwata et al, 1995; Pereira et al, 2001; Svensson-Ek et al, 2002) and consequently their variation provide complementary molecular information regarding changes in the primary sequence of the COX1 protein, upon which phylogenetic trees are reconstructed (Degli Esposti et al, 2019; Ducluzeau et al, 2014; Golyshina et al, 2016; Han et al, 2011; Hemp and Gennis, 2008; Pereira et al, 2001; Sousa et al, 2012; Spang et al, 2019). To confirm this, we verified that the tree topology of *Acidithiobacillus* COX1 is not significantly modified upon substituting the non-ionizable residues present in its modified K-channel (Supplementary Figure S7) with those typical of the channel such as the eponymous K354 of *P. denitrificans* (Iwata et al, 1995). Moreover, the COX1 proteins of the CyoCAB subclade (Degli Esposti et al, 2019) present very different K-channel configurations, which are classified under different categories (Supplementary Figure S7), despite their strong sequence similarity. To expand earlier surveys (Degli Esposti, 2020; Degli Esposti et al, 2020), we introduced here an enlarged classification of K-channels that can resolve previously ambiguous combinations of amino acid residues using the additional 'hybrid' category, as well as the protonable nature of the single residue of the COX2 subunit that seems to form the entry to the proton channel (Pereira et al, 2001), as shown in Supplementary Figure S7. While the substitution of such a residue by a non-ionizable amino acid often correlated with the loss of the 'canonical' K-channel originally found in *P. denitrificans* COX, in some cases it did not, thereby providing additional information for the classification of K-channel variants (Supplementary Figure S7).

We systematically analyzed also the presence of additional TM at the N terminus of COX1 (which normally has 12 TM), abbreviated as 'TM extra' in Supplementary Table S1. This molecular feature appears to have ancestral character in COX1 proteins, since it is shared with several family C paralogs (Degli Esposti, 2020) and consequently can contribute information for disentangling COX phylogeny. The most common feature of 2 additional TM at the N-terminus, abbreviated as '2TM extra', is mentioned in the main text with regard to the molecular phylogeny of archaeal COX, since it is present in

COX1-3 fusion proteins of *Ca.* Marsarchaeota (Figure 5). In phylogenetic trees of bacterial COX1, the presence of the '2TM extra' feature provided a new way to systematically label proteins that are usually classified as type A2, even if they have the molecular signature PEVY in TM6 that is normally associated with type A1 oxidases (Pereira et al, 2001; Sousa et al, 2012). The situation often applied to COX1 proteins from acidophilic Proteobacteria such as *Acidithiobacillus* spp. and acidophilic Firmicutes such as *Sulfobacillus* spp., as well as to a variety of deep branching COX1 from soil or aquatic environments (Degli Esposti, 2020). Hence, phylogenetic trees have been predominantly constructed by including full length COX1 sequences, thus maintaining the N-terminal part that was routinely trimmed in previous studies (Degli Esposti et al, 2019; Ducluzeau et al, 2014; Golyshina et al, 2016; Han et al, 2011; Hemp and Gennis, 2008; Pereira et al, 2001; Sousa et al, 2012; Spang et al, 2019) and also Supplementary Fig. S8A. Given that the 2TM extra feature is shared with several FixN paralogs of the family C (Degli Esposti, 2020; Pereira et al, 2001) but not family B paralogs, subsequent phylogenetic trees included only family C proteins as the outgroup (Figure 6A and Supplementary Figures S9-S12).

The two major topologies of extended COX1 trees are shown in Supplementary Figures S9A and S9C and were evaluated with integrated analysis of the ancestral features described above as in Figure 5. Mapping the various categories of the K-channel on the Bayesian tree of Supplementary Figure S9A led to the following evolutionary implications. The 'canonical' K-channel might have evolved either in the common ancestor of the type A1 clade in which it is widespread (and then distributed to other COX1 by LGT), or earlier in the common ancestor of both major clades. In the latter scenario, the ancestral form of the 'canonical' K-channel would have been lost at least six times by different modifications along COX evolution, even multiple times within individual subclades (particularly that of CyoCAB at the bottom of the tree). The former scenario would be more conservative regarding the evolution of the 'canonical' K-channel because it could explain its presence in subclades of type A2, for example the subtype a-I, by LGT events to their common ancestor. However, the same scenario would hardly explain the presence of K^C-channel variants in three subclades of the type A1, for instance in that of Elusimicrobia (Supplementary Figures 7 and 9A).

Conversely, mapping the various categories of the K-channel onto the ML tree (Supplementary Figure S9C) suggested a discrete evolution of the 'canonical' K-channel from an ancestral K^C-channel (or hybrid) shared with the common ancestor of family C paralogs at the node indicated by the red arrow in Figure S9C. The common ancestor represented by this node would have possessed a hybrid form of the K-channel that could mutate into either a 'canonical' K-channel or a K^C-channel-like by a few amino acid

changes, consistent with the variation in channel-forming protonable amino acid residues (Supplementary Figure S7). In other words, the plasticity of the COX1 protein (Ducluzeau et al, 2014) and the integrated evolution of its functionally related residues that are involved in proton pumping (Degli Esposti, 2020) may have led to sequential variants of the K-channel along COX evolution, without involving multiple events of LGT across taxonomically separate lineages of bacteria. The topology of the ML tree in Supplementary Figure S9C, therefore, looks more plausible than the topology displayed by the tree in Supplementary Figure S9A.

Mapping the presence of the 2TM extra feature on phylogenetic trees provided further support to the above conclusion, since it implied a single loss rather than two or more losses along COX evolution (Supplementary Figure S9C). However, trees such as that in Supplementary Figure S9C could hardly represent the most likely phylogeny of COX1 proteins, since they include branches with undesired features leading to artifacts, as mentioned in connection to the subclades of Solirubrobacterales. A second approach to alignment building and refinement was thus necessary for better resolving the phylogeny of bacterial COX1.

A second approach to resolve COX1 phylogeny

Usually, the alignment of multiple COX1 protein sequences for reproducing phylogenetic trees were built by computer programs first (see Materials and Methods), which invariably introduce many gaps to maximize local sequence similarity. Such gaps were reduced by subsequent manual refinements (Degli Esposti et al, 2019; Ducluzeau et al, 2014; Hemp and Gennis, 2008; Sousa et al, 2012). In contrast, our new approach started by aligning *ca.* 20 COX1 sequences, mostly possessing the 2TM extra feature, with a few FixN paralogs, which introduced a reduced set of gaps to match local sequence similarity after manual refinement of an initial alignment obtained with the MUSCLE program (Supplementary Figure S10). The close reproducibility of phylogenetic trees obtained with different programs was verified before adding other COX1 sequences to the alignment (Supplementary Figure S10). Such additional sequences were added up to a total of about 50 without inserting new gaps. Inserts that would be required to maximize the match of additional sequences were not considered in expanding the alignment. These alignments provided maximal representation of the recognized subclades of bacterial COX1, after excluding those exhibiting clear deviations from average values of branch length or substitution rate.

Subsequently, detailed analysis of Bayesian BEAST trees obtained with these COX1 sets unveiled the presence of subclades with branches significantly longer than average, as shown in Supplementary Figure

S11B. The proteins of such branches were replaced and the analysis of branch length was undertaken again, revealing that COX1 of type A1 subtype a-III exhibited a branch that was significantly longer than average, thereby potentially reducing the strength in posterior support of an internal node of the branch containing type A1 COX1 (Supplementary Figure S12). The substitution of just one of the subtype a-III sequences that were originally present with that of another A1 subtype was sufficient to strengthen the support of this internal node and the overall stability of the Bayesian tree of 40 COX1 proteins (Figure 7, cf. Supplementary Figure S12). Thus, trees such as that shown Figure 7A could represent a reproducible robust phylogeny for bacterial COX1 exhibiting maximal overall support.

Analysis of CtaG proteins: caa3_CtaG

Phylogenetic trees of diverse caa3_CtaG proteins, the distribution of which is reported in Supplementary Table S2, have indicated partition into three major clades, as shown in Figure 8. Clade 1 is probably the largest, including proteins from the phyla of Proteobacteria, Chloroflexi and Actinobacteria, in particular the bi-functional proteins of *Corynebacterium* and related taxa (Supplementary Figures S13 and S14). Clade 2 characteristically contains proteins from taxa of the *phylum* Firmicutes such as *B. subtilis*, but is taxonomically very diverse. Indeed, it additionally includes caa3_CtaG proteins of Gemmatimonadetes, Chloroflexi (especially of the family Ktenobacteraceae), acidophilic Actinobacteria such as *Acidithrix*, *Ca.* Dadabacteria and *Ca.* Entotheonella. Three proteins of *Ca.* Melaniabacteria closely cluster together with those of *Ca.* Entotheonella (Supplementary Figures S13 and S14) within the same clade, a likely result of LGT since *Ca.* Melainabacteria do not contain heme A-containing oxidases (Soo et al, 2017). The third clade is early branching and exclusively formed by caa3_CtaG proteins of Actinobacteria living in soil environments such as Solirubrobacterales and Thermoleophilaceae (Supplementary Figures S13 and S14). One representative of this clade, an Actinobacteria MAG, was previously found to branch early in unresolved phylogenetic trees (Supplementary Figure S13A), cf. (Degli Esposti et al, 2020). Finally, the highly divergent caa3_CtaG orthologs of iron-oxidizing *Acidithiobacillus* spp. and *Acidiferrobacter* spp. form basal sub-branches in ML trees, as well as a single clade in Bayesian trees (Figure 8B and Supplementary Figures S13 and S14), thus confirming our previous results (Degli Esposti et al, 2020).

Larger alignments than that used for generating the tree in Figure 8B were progressively purified of long branches (Supplementary Figures S13B and S14A) on the basis of the 95% confidence range of branch length values determined by the BEAST program, as described before for CtaA and COX1. Additionally, the weak posterior support for a branch in major clade 1 (orange circle in Supplementary Figure S14A)

was enhanced by replacing several caa3_CtaG sequences that still exhibited long branches with those that showed close to average branch length and clustered in the same clade, thereby obtaining acceptable values of support: above 76% in the Bayesian tree (Figure 8B), and over 80%Ultrafast bootstraps in the ML tree for the same alignment of caa3_CtaG proteins (Supplementary Figure S14B). This enhanced support for clade 1 was obtained without significant change in the overall topology of the phylogenetic trees, thereby indicating good reproducibility in evaluating the molecular phylogeny of caa3_CtaG proteins, despite the low number of amino acid sites and poor sequence identity (only one residue was invariant in the alignments used here, as mentioned in the main text).

Analysis of CtaG proteins: CtaG_Cox11

In the main text we have focused on the caa3_CtaG family of Cu chaperons that are required for the assembly of Cu_B in nascent COX1, because such proteins are present in iron oxidizers and other taxa that possess deep branching COX (Supplementary Tables S1 and S2). Here we present also the results of our phylogenetic analysis of the other family of CtaG, which is homologous to mitochondrial Cox11 and fulfills the above function in eukaryotes (Banci et al, 2004; Degli Esposti et al. 2019). This protein is part of the CtaG_Cox11 family that originated in Proteobacteria and is predominantly present in alphaproteobacteria as part of the A1 type COX operon subtype ab, generally in sinteny with COX3 (Degli Esposti et al, 2019). Structurally, it has only one TM and its catalytic site is exposed to the outer face of the cytoplasmic membrane of bacteria, or of the inner mitochondrial membrane in eukaryotes (Banci et al, 2004; Degli Esposti et al, 2019). The absence of recognized paralogs, the restricted distribution to alphaproteobacteria and the small size of about 200 aa render the phylogenetic analysis of CtaG_Cox11 quite challenging. Fortunately, we found distant relatives in deep branching Proteobacteria such as Magnetococcales MAGs (Supplementary Table S2) that provide a reasonable outgroup to root the phylogenetic trees, which were extended to COX11 proteins from diverse supergroups of eukaryotes (Burki et al, 2020) that are currently available in the nr database (Supplementary Fig. S15). No homologs could be found in Archamoebae, though. Cox11 proteins from Archaeplastida (red and green algae) appear to form the deepest branches in the monophyletic clade of eukaryotic Cox11 (Supplementary Fig. S15). Intriguingly, the bacterial sister clade to this eukaryotic clade consistently contains CtaG_Cox11 proteins from Holosporales, including Alphaproteobacteria bacterium 41_28 (Supplementary Fig. S15 and data not shown - see also Supplementary Table S2), which has been recently associated with this order of alphaproteobacteria (Degli Esposti et al, 2019b). Various members of the polyphyletic Rhodospirillaceae family, now subdivided in diverse taxonomic entities as indicated in Supplementary

Table S2 (Degli Esposti et al, 2019b; Parks et al, 2018), form branches subtending the above sister clades, in diverse order depending upon the phylogenetic inference and model used (Supplementary Fig. S15 and data not shown). Therefore, it is currently difficult to define probable alphaproteobacterial ancestors for mitochondrial COX11 on the basis of the available proteins and genomes.

Statistical analysis of phylogenetic trees

Phylogenetic analysis of COX1 included quantitative evaluation of the length and substitution rate of each branch as performed for CtaA (cf. Supplementary Figures S5 and S8b). We realized that the pervasive presence of long branches in COX1 trees demanded a thorough statistical analysis of the parameter of branch length to limit the artifacts arising from LBA and unequal rates of substitution in the phylogenetic trees. Rather than using the common 'inspection' of long branches in preliminary phylogenetic trees, for example (Spang et al, 2019), we undertook quantitative analysis of the length and substitution rate of tree branches following two approaches. In the case of ML trees, these branch parameters could be obtained as raw values using the FigTree program and were therefore compared across several trees obtained with the same program and alignments of similar size (Fig. 6). The statistical significance of deviations from the average were determined using the non-parametric test of Mann-Whitney with 95% confidence using the MiniTab19 program https://www.minitab.com/en-us/products/minitab/ ; p values <0.05 were considered statistically significant. An equivalent analysis was undertaken with the median values of either branch length or branch substitution rate that were obtained from different BEAST Bayesian trees.

The second approach exploited the statistical resources of the BEAST program, which can elaborate not just the median values, but also the 95%_HPD (High Posterior Density) interval of the values for the above branch parameters. Such values are the Bayesian equivalent to the 95% confidence interval of other statistical methods (Drummond and Bouckaert, 2015). To verify whether the branch length of a given subclade in CtaA or COX1 trees was significantly different from the average length of subclades containing structurally similar proteins in the same Bayesian tree, we often used the shortcut of statistically evaluating the bottom (low) values of the 95%_HPD intervals. Empirically, such values were found to be significantly larger than the average branch length when they exceeded the median plus three Standard Deviations (3 SD) of the subclades containing structurally similar proteins, as previously reported for related statistical analyses (Degli Esposti et al., 2019b). For instance, in the case of the Bayesian CtaA tree of Supplementary Figure S1A, the median of the bottom 95%_HPD values of three

subclades of type 1 CtaA was 0.3 with a SD of 0.12, hence the median plus 3D reference value was 0.66 (red histogram in the middle of Supplementary Figure S1C). The bottom 95%_HPD value of the *Thermus* subclade of fused CtaA proteins (1.31) and that of the short CtaA proteins from archaea (1.06 for type 1.4 CtaA of either *Aeropyrum* or *Halobacterium* spp.) were clearly higher than 0.66 (Supplementary Figure S1C) and consequently deemed to have a branch length significantly larger than average. In the case of the *Thermus* subclade, the bottom (low) 95%_HPD value was also larger than the top 95%_HPD values of other type 1 CtaA and their median values.

Independent confirmation of the validity of our statistical analysis emerged by comparison with previously reported results of branch substitution rate. In our Bayesian BEAST trees of COX1, the orthologs from Verrucomicrobia such as *Methylacidiphilium* spp. displayed values of median branch rate that were significantly larger than average (Supplementary Table S1), in agreement with previous results reported for methane monooxygenase proteins of the same taxa (Khadka et al, 2018). For this reason, *Methylacidiphilium* COX1 was not considered further in our phylogenetic analyses. Conversely, the COX1 proteins of Cyanobacteria generally displayed a median branch substitution rate that was significantly lower than average, in agreement with previous reports indicating a slow rate of substitution in membrane redox proteins of Cyanobacteria (Cardona et al, 2019; and references therein). Despite this slow substitution rate, removing cyanobacterial COX1 had little effect on the topology of phylogenetic trees. Therefore, representative cyanobacterial COX1 proteins were generally maintained in our analyses.

References cited only in the Supplementary Material

Ahn Y. O., Albertsson I., Gennis R. B., Ädelroth P. Mechanism of proton transfer through the K(C) proton pathway in the Vibrio cholerae *cbb*(3) terminal oxidase (2018). *Biochim Biophys Acta* 1859, 1191-1198.

Banci, L., Bertini, I., Cantini, F., Ciofi-Baffoni, S., Gonnelli, L., Mangani, S. Solution structure of Cox11, a novel type of β-immunoglobulin-like fold involved in CuB site formation of cytochrome c oxidase (2004). *J Biol Chem* 279, 34833-34839.

Brigandt I., Love, A. (2017). "Reductionism in Biology". In Zalta, Edward N. (ed.). Stanford Encyclopedia of Philosophy. Metaphysics Research Lab, Stanford University, Ca USA.

Burki, F., Roger, A.J., Brown, M.W., Simpson, A.G. The new tree of eukaryotes (2020). *Trends in ecology & evolution* 35, 43-55.

Cardona T., Sánchez-Baracaldo P., Rutherford A. W., Larkum A.W. Early Archean origin of Photosystem II (2019). *Geobiology* 17, 127-150.

Degli Esposti, M., Lozano, L., Martínez-Romero, E. Current phylogeny of Rhodospirillaceae: A multi-approach study (2019b). *Molecular Phylogenetics and Evolution* 139, 106546.

Kozubal M. A., Dlakic M., Macur R. E., Inskeep W. P. Terminal oxidase diversity and function in "Metallosphaera yellowstonensis": gene expression and protein modeling suggest mechanisms of Fe(II) oxidation in the sulfolobales (2011). *Appl Environ Microbiol* 77, 1844-1853.

Lewin A., Hederstedt L. Compact archaeal variant of heme A synthase (2006). *FEBS Lett* 580, 5351-5356.

Hu D., Zang Y., Mao Y., Gao B. Identification of Molecular Markers That Are Specific to the Class Thermoleophilia (2019). *Frontiers Microbiol* 10, 1185.

Ren H., Ma H., Li H., Huang L., Luo Y. Acidimangrovimonas sediminis gen. nov., sp. nov., isolated from mangrove sediment and reclassification of Defluviimonas indica as Acidimangrovimonas indica comb. nov. and Defluviimonas pyrenivorans as Acidimangrovimonas pyrenivorans comb. nov (2019). *Int J. Sys Evol Microbiol* 69, 2445-2451.

Sousa F. L., Alves R. J., Ribeiro M. A., Pereira-Leal .J B., Teixeira M., Pereira M. M. The superfamily of heme-copper oxygen reductases: types and evolutionary considerations (2012). *Biochim Biophys. Acta* 1817, 629–637.

Uyeda J. C., Harmon L. J., Blank C. E. A. Comprehensive Study of Cyanobacterial Morphological and Ecological Evolutionary Dynamics through Deep Geologic Time (2016). *PloS One* 11, e0162539.

Wang Z. O., Pollock D. D. Coevolutionary patterns in cytochrome *c* oxidase subunit I depend on structural and functional context (2007). *J Mol Evol* 65, 485-495.

List of Supplementary Table and Figures

Table S1. Major subclades of COX1 proteins in prokaryotes. The table is provided also as an Excel file.

Table S2. Distribution of CtaG proteins in bacterial subclades and eukaryotic supergroups.

Figure S1. CtaA phylogenic analysis presented prior to this work.

Figure S2. IQ-Tree consensus ML trees for CtaA.

Figure S3. Phylogenetic trees of 95 CtaA protein sequences without archaeal and DUF420 proteins.

Figure S4. Branch variation in phylogenetic trees of 100 bacterial CtaA proteins.

Figure S5. BEAST MCC trees obtained with an alignment of 100 CtaA and DUF420 proteins and statistical analysis of median branch length of several clades.

Figure S6. Taxonomic distribution of type 0 CtaA.

Figure S7. Amino acid variation of the K-channel in bacterial COX1 and COX2.

Figure S8. Extended phylogenic tree of COX1 from bacterial and Archaeal taxa.

Figure S9. Phylogeny of bacterial COX1.

Figure S10. ML trees of 22 COX1 and 2 FixN sequences.

Figure S11. Branch length evaluation in a robust Bayesian tree of 48 COX1 proteins.

Figure S12. Panel a shows a Bayesian BEAST tree obtained from an alignment of 40 COX1.

Figure S13. Comparison of ML trees of caa3_CtaG proteins.

Figure S14. Bayesian BEAST tree of 44 caa3_CtaG proteins and an IQ-Tree ML tree.

Figure S15. ML tree of 46 eukaryotic and bacterial CtaG_Cox11 proteins.

Supplementary Table S1. **Major subclades of COX1 proteins in prokaryotes.**

Supplementary Table S1.xls

**bacteria**							
**general definition of subclade**	**taxa**	**2TM extra**	**K-channel**	**mutation rate**	**branch lengh**	**caa3_CtaG**	**NOTES**
**A family, A1 type**							
**subtype (a)b**	AlphaBetaGammaProteobacteria	**no**	**yes**	average	average	**no**	
subtype a & a-II COX1 only	Proteobacteria, Chloroflexi, other taxa	**no**	**yes**	fast		**yes, Table S2**	
subtype a-III (2 COX3)	Proteobacteria, Acidobacteria, other taxa (conserved cassette), CFB (split)	**no**	**yes**	average	average	**no**	
ctaA-G/caa3	Bacilli (Firmicutes) & Chloroflexi	**no**	**yes**	average	average to long	**yes, Table S2**	
Gemmatimonadetes ★	Gemmatimonadetes, Acidobacteria, *Ca.* Entotheonella, *Ca.* Rokubacteria, *Ca.* Poribacteria, other taxa	**no**	generally yes	some fast, most average	some long	**yes Table S2**	
new operon Chloroflexi	Chloroflexi MAG (soil metagenomes)	**no**	**yes**	fast	long	**no**	
Oligoflexia	*Bacteriovorax* and other taxa	**no**	**no, KC**	fast	long	**no**	
KC channel	Elusimicrobia, *Ca.* Calescamantes, Methylacidiphylaceae (Verrucomicrobia)	**no**	**no, KC**	fast or average	**long**	**no**	
bo3 ubiquinol oxidases	Proteobacteria, Firmicutes, Chloroflexi, Actinobacteria, other taxa	**no**	**yes**	**fast**	**long**	**no**	
subtype a COX13 fused	Proteobacteria, a few Chloroflexi	**no**	**yes**	fast	**long**	some	
**A family, A2 type**							
Thermus COX13 fused	Deinococcus-Thermus	**no**	**yes**	fast	**long**	some isolated	
Planctomycetian	PVC, *Ca.* Omnitrophica, Acidobacteria, other taxa	**no**	**yes**	fast	variable	**no**	classified as A2 type but clustering with A1 type ★ Gemmatiminadetes
subtype a-I (± Act)	Proteobacteria, Bdellovibrionales (Oligoflexia), *Ca.* Dadabacteria, *Ca.* Zixibacteria, Leptospirales	**no**	**yes**	generally average	average to long	**no**	
subtype delta	Epsilon and Deltaproteonacteria, Ignavibacteria	**no**	**yes**	average	average	**no**	
subtype CyoCAB	Aquificae, Zetaproteobacteria, AlphaBetaGammaDeltaProteobacteria, Nitrospirae, other unclassified taxa	**no**	some **yes**, **most KC, hybrid or modified**	fast, especially Aquificae and Zetaproteo.	average or long	**no**	
Cyanobacteria CyoBAC	Cyanobacteria	**no**	**yes**	**slow**	average	2 by LGT	
Solirubrobacterales	Actinobacteria	**yes, 2**	**yes**	fast	**long**	**yes Table S2**	
Anaerolinae	Chloroflexi, especially unclassified & Anaerolinae	**yes, 3 to 0**	**yes**	fast	**long**	some	
Chloroflexi hybrid/KC channel	unclassified Chloroflexi, Elusimicrobia, unclassified Actinobacteria	**yes, 2 or 1**	**no, KC or hybrid**	fast	**long**	**yes Table S2**	
2TM Chloroflexi & Actinobacteria	Chloroflexi, Acidimicrobia and other Actinobacteria, *Ca.* Dormibacter	**yes, 2**	**no, KC, hybrid or modified**	slow	average	**yes Table S2**	HPEVY motif TM6 but classified as A2 type
2TM acidophilic Firmicutes	*Alicyclobacillus*, *Sulfobacillus* & *Acidibacillus* - *Thermoaerobacter*	**yes, 2**	**no, modified**	fast	average to long	**yes Table S2**	HPEVY motif TM6 but classified as A2 type
2TM *Thioclava*	unclassified *Thioclav* a (alphaproteobateria)	**yes, 2**	**no, modified**	fast	**long**	**yes Table S2**	HPEVY motif TM6 but classified as A2 type
2TM acidophilic Proteobactera	*Acidihalobacter*, *Acidiferrobacter*, Acidithiobacillaceae	**yes**	**no, modified**	fast or average	average to long	**yes Table S2**	HPEVY motif TM6 but classified as A2 type
**archaea**				relative to archaea only			
**general definition of subclade**	**taxa**	**2TM extra**	**K-channel**	**mutation rate**	**branch length**		**NOTES**
**A family, A2 type**							
Åsgard COX1	*Ca.* Heimdallarchaeota, unclassified Åsgard	**no**	**yes**	average	average	**no**	deepest branching clade 1 in Fig. 5
**A family, A1 type**							
Crenarchaeota COX13	Sulfolobales, *Pyrobaculum* (Thermoprotei	**no**	**no, hybrid**	average	**long**	related DUF1404	fused with COX3
Halobacteria COX1	most Halobacteriales	**yes, 1**	**yes**	average	average	**no**	A1 type, deepest branching clade 2
Ca. Marsarchaeota COX13	*Ca.* Marsarchaeota	**yes, 2**	**no, modified**	average	average	**no**	fused with COX3
Haloarcula hispanica COX13	some Haloarculaceae (Halobacteria)	**yes, 1**	**no, hybrid**	average	average	**no**	fused with COX3
Aeropyrum COX13	many Desulfurococcales (Thermoprotei)	**no**	**no, modified**	average	long	**no**	fused with COX3
Thermoplasmatales COX13	Ferroplasmataceae (Thermoplasmatales), one or more unclassified archaea	**no**	**no, modified**	average	average to long	**no**	fused with COX3
Euryarchaeota COX1 simple operon	unclassified Euryarchaeota (marine metagenomes), Diaphorachaeota	**no**	**yes**	average	average to long	**no**	A1 type, cluster with in a clade of Proteobacteria, especially betaproteobacteria
**B family**							
Cuniculiplasmataceae	Cuniculiplasmataceae, unclassified Thermoplasmatales	**yes, 1**	**yes**	fast	**long**	**no**	lacks COX3, hybrid with A family
SoxB	Sulfolobales, some Thermoprotei	**no**	**yes**	fast or average	**long**	**no**	
DoxB	Sulfolobales	**no**	**yes**	fast	**long**	**no**	
FoxA	iron-oxidizing Sulfolobales	**no**	**yes**	fast	**long**	**no**	

KC, K^C channel typical of C family oxidases, but present also in some family A oxidases (Figure S7).

Supplementary Table S2. Distribution of CtaG proteins in bacterial subclades and eukaryotic supergroups. The two different families of Cu-assembly proteins CtaG are listed following the COX1 subclades for bacterial A family oxidases listed in Supplementary Table S1 and also, in the case of CtaG_Cox11, according to the recent classification of eukaryotic lineages and supergroups (Burki et al, 2020), following the phylogenetic pattern and subclades observed in phylogenetic trees such as that of Supplementary Fig. S15.

Revised Supplementary Table S2CtaG.xls

**Supplementary Table S2**			
**caa3_CtaG**			
**general definition of subclade**	**bacterial lineage**	**caa3_CtaG representatives and taxa**	**NOTES**
subtype a	Proteobacteria, other taxa	PZN04705 Proteobacteria_ZC4RG42; WP_085083811 Azospirillum_oryzae; WP_007435270 Acetobacteraceae_AT-5844; WP_114162484 Paraburkholderia_terricola. 6TM: WP_082828323 Tistrella_mobilis. Rhodovibrio group: WP_081728717 Rhodovibrio_salinarum; THD10598 Metallibacterium_scheffleri;	Clade 1
ctaA-G/caa3	Bacilli (Firmicutes)	WP_044427878 Bacillus_subtilis	Clade 2, model in Fig. 8A
Gemmatimonadetes	Gemmatimonadetes, Acidobacteria, *Ca.* Entotheonella, *Ca.* Rokubacteria, other taxa including Ca. Melainabacteria	WP_089722083 *Ca.* Entotheonella_palauensis; PKL79953 *Ca.* Melainabacteria bacterium HGW-Melainabacteria-1; OLC37546 Gemmatimonadetes_13_1_40CM_4_65_7; MBI3626227 *Ca.* Rokubacteria_NC_groundwater_967_Pr1 (metagenome)	Clade 2
Thermus COX13 fused	Deinococcus-Thermus	WP_051307968 Deinococcus_ficus	isolated from COX gene cluster
Cyanobacteria CyoBAC	Cyanobacteria	WP_146007319 Fischerella_thermalis (partial)	most likely due to LGT
Anaerolinae	Anaerolinae	MAU11883 Anaerolineaceae_NAT117	rare
Chloroflexi hybrid/KC channel	unclassified Chloroflexi	KRT64361 Chloroflexi_CSP1-4	Clade 1
2TM Chloroflexi & Actinobacteria	Acidimicrobia and other Actinobacteria, *Ca.* Dormibacter	KJF18403 Acidithrix_ferrooxidans; PZR82374 *Ca.* Dormibacter sp. RRmetagenome_bin12	Clade 2
2TM acidophilic Firmicutes	*Sulfobacillus* & *Acidibacillus*	WP_053960858 Sulfobacillus_thermosulfidooxidans; PSR35265 Sulfobacillus_benefaciens	Clade 2
Solirubrobacterales	Actinobacteria	OJU82991 Solirubrobacterales_70-9 ; WP_146918550 Baekduia_soli; MBA2420105 Thermoleophilaceae_MGR_bin235	Clade 3
2TM acidophilic Proteobactera	*Acidihalobacter, Acidiferrobacter,* Acidithiobacillaceae	WP_163096436 Acidithiobacillus_ferrianus; WP_083995572_Acidiferrobacter_thiooxydans	basal clade, model in Fig. 8C
**CtaG_Cox11**			
**general definition of subclade**	**bacterial lineage - eukaryotic supergroup**	**Cox11 representatives and taxa**	**NOTES**
**bacteria**			
subtype a	Proteobacteria, other taxa	MAF31647_Magnetococcales_ARS4	unique insert, basal
subtype ab	Alphaproteobacteria	Geminicoccaceae: WP_035484761 Geminicoccus roseus; WP_088560775 Arboricoccus pini. Rhodospirillales (various taxonomic entities): WP_014746325 Tistrella mobilis; EME68549 Magnetospirillum caucaseum; QNT71357 Defluviicoccus vanus; WP_094409708 Elstera cyanobacteriorum; WP_144258743 Ferrovibrio terrae; WP_189045690 Aliidongia dinghuensis; WP_015466638 Micavibrio aeruginosavorus; WP_044433212 Skermanella aerolata. TMED109 order of marine MAGs: OUV28667 Alphaproteobacteria_TMED109; PPR17067 Alphaproteobacteria_MarineAlpha9_Bin3. Holosporales: OJX08829 Caedibacter sp. 38-128; WP_010297984 *Ca* . Odyssella thessalonicensis; WP_085784321 *Ca.* Nucleicultrix amoebiphila; OJW51488 Alphaproteobacteria 41-28.	Holosporales form the sister clade to the clade of mitochondrial Cox11
**eukaryotes**			
Archaeplastida	red algae, green algae, Haptophyta and Acanthamoeba - derived Amoebozoan	Rhodophyta: BAM79589 Cyanidioschyzon merolae; CDF35956 Chondrus crispus; EME30450 Galdieria sulphuraria. Green algae: ACO60923 Micromonas commoda; PNW89051 Chlamydomonas reinhardtii. Haptophyta: KOO34120 Chrysochromulina tobinii. Amoebozoa: XP_004335469 Acanthamoeba castellanii.	deep branching
Cryptpophyta	Cryptpophyta	EKX46966 Guillardia theta	
Euglenozoa & SAR	Jakobida, SAR (Rhizaria)	AGH23975 Andalucia godoyi; AGH24265 Reclinomonas americana; BBD14124 Ophirina amphinema. Rhizaria: CEO98488 Plasmodiophora.	
Amoebozoa & Orphans	Amoebozoa, Colliodyctionidae and Apusozoa	Amoebozoa: NDV36711 Arcella; XP_003294442 Dictyostelium_purpureum. Collodictyonidae: BAU71458 Diphylleia. Apusozoa: KNC55610 Thecamonas trahens	
Amoebozoa & Animalia	Amoebozoa, Filasterea and Metazoa	Amoebozoa: PRP78593 Planoprotostelium. Filasterea: KJE97994 Capsaspora. Metazoa: CDG66245 Hydra; GAU87765 Ramazzottius_Tardigrades.	late branching

Supplementary Figure S1. **CtaA phylogeny analysis prior to this work.**

Panel a. Bayesian MCC tree of 40 CtaA protein sequences. Posterior support is proportional to branch width and annotated in percent values, red when equal or below 50%. The total number of amino acid sites was 350. **Panel b**. Bayesian MCC tree as in Panel a with median branch length. The short CtaA of the archaean *Aeropyrum pernix* is highlighted. In ML trees obtained with the MEGA and IQ-Tree program this protein clusters with the short protein of *Halobacterium* sp., which is fused with CtaB. **Panel c**. Quantitative evaluation of branch length from the tree in Panel b. The bottom values of the 95%_HPD (High Posterior Density, equivalent to confidence interval - Drummand and Bouckaert, 2015) branch length are in histogram form, with the central red bar representing the median bottom level plus 3SD. All the bars on the right are higher, thus showing high statistical probability of being branches longer than average.

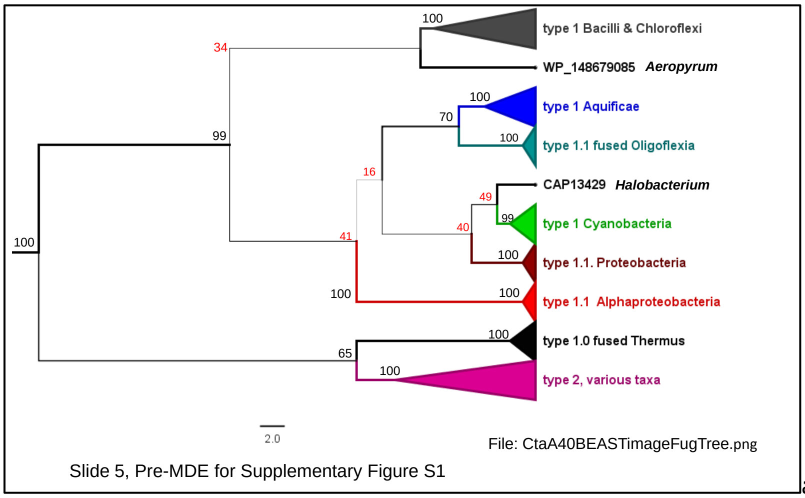

a

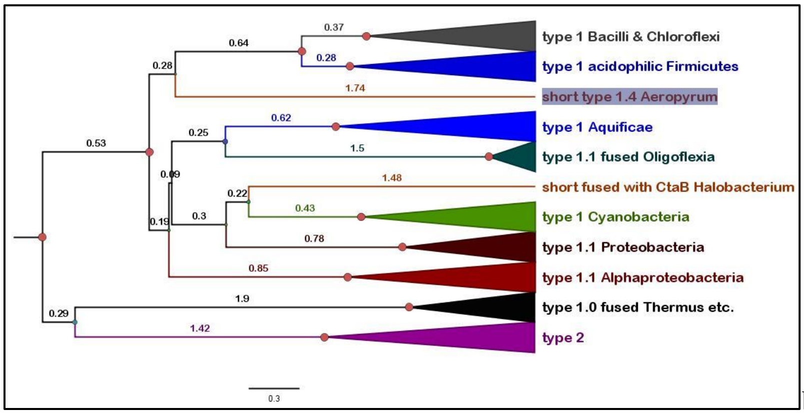

b

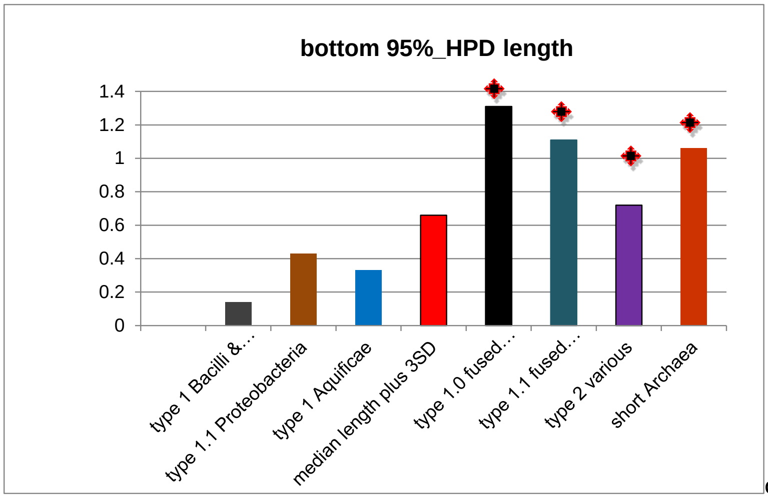

c

Supplementary Figure S2. IQ-Tree consensus ML trees for CtaA.

Panel a. IQ-Tree consensus ML tree obtained with the same alignment of 60 CtaA proteins as in Figure 3A using the LG model. The total number of amino acid sites in the alignment was 358. **Panel b.** IQ-Tree consensus ML tree obtained with the same alignment and the mixture model EX_EHO (Le et al, 2010). Note the stronger % values of Ultrafast bootstrap support.

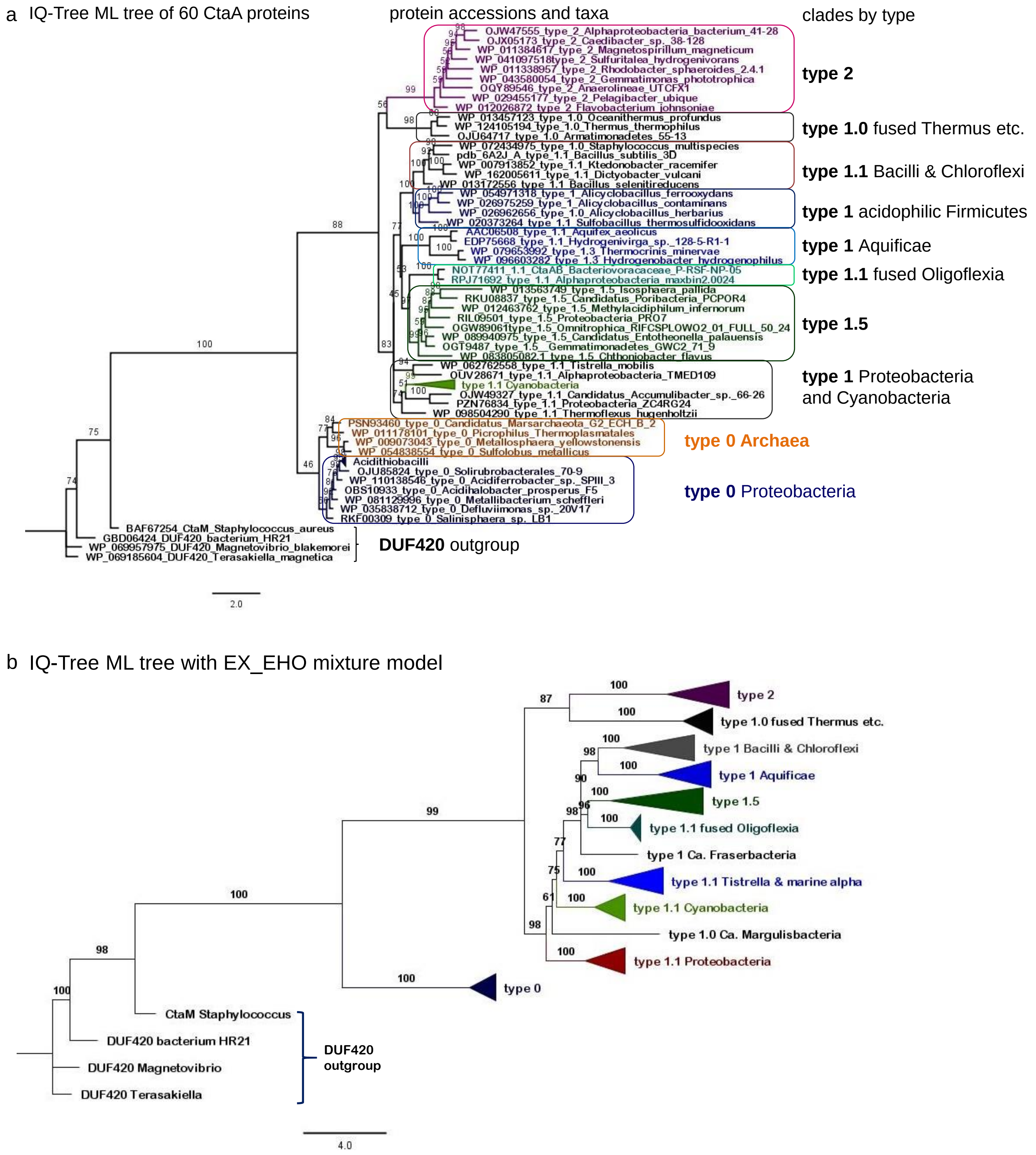

Supplementary Figure S3. Phylogenetic trees of 95 CtaA sequences without archaeal CtaA and DUF420 proteins.

Bayesian MCC tree produced with the BEAST program using a refined alignment of 94 bacterial CtaA proteins and the unusual Cox15 of type 1.1 coded by the mtDNA of *Andalucia* (He et al, 2016). The alignment included type 1.0 proteins of Verrucomicrobia and marine MAGs that have been recently deposited in Genbank that show the conservative substitution of otherwise invariant E57 (*B. subtilis* CtaA numbering [Niwa et al, 2018]; Table 1) and are presumed to be functional since no other heme A synthase is present in their genomes containing family A COX. The tree has been used as the basis for the model of CtaA molecular evolution in Figure 3B. Posterior values of branch support are in decimals, and are not as strong as in other Bayesian trees (e.g. Figure 3) because of the very limited number of invariant residues (Degli Esposti et al, 2019) and the large sequence variation exhibited by deduced CtaA sequences from MAGs. The alignment included 355 amino acid sites. *Andalucia* Cox15 clusters in a clade containing various type 1.1 proteins of marine alphaproteobacteria including those of the TMED109 order (see Supplementary Table S2), confirming previous results (Degli Esposti et al, 2020).

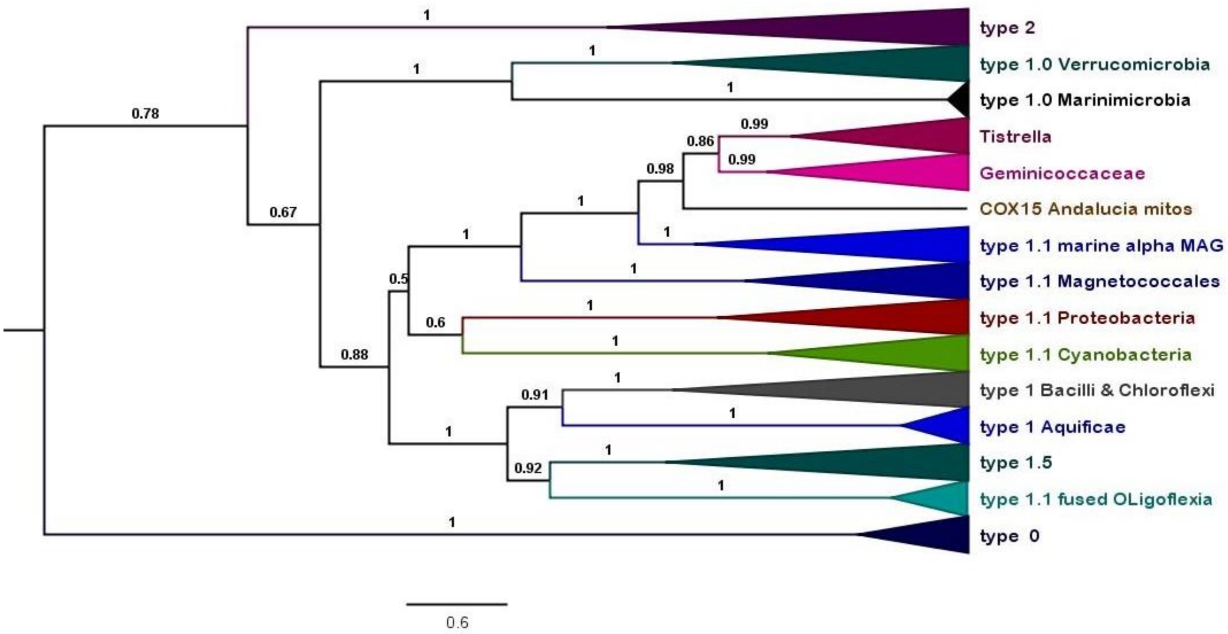

Supplementary Figure S4. Branch variation in phylogenetic trees of 100 bacterial CtaA proteins. **Panel a.** The IQ-Tree consensus ML tree was obtained with the mixture substitution model C20 (He et al, 2016) to improve the robustness of phylogenetic trees obtained with different types of CtaA proteins for a total of . **Panel b.** The IQ-Tree consensus ML tree was obtained with the same WAG model used for the Bayesian tree in Supplementary Figure S3. The trees in both panels were reconstructed from an alignment of 99 bacterial protein plus the Cox15 of *Andalucia* mitochondria that was slightly expanded form that used in Supplementary Figure S3 and contained 358 amino acid sites. Values represent %Ultrafast bootstraps.

a

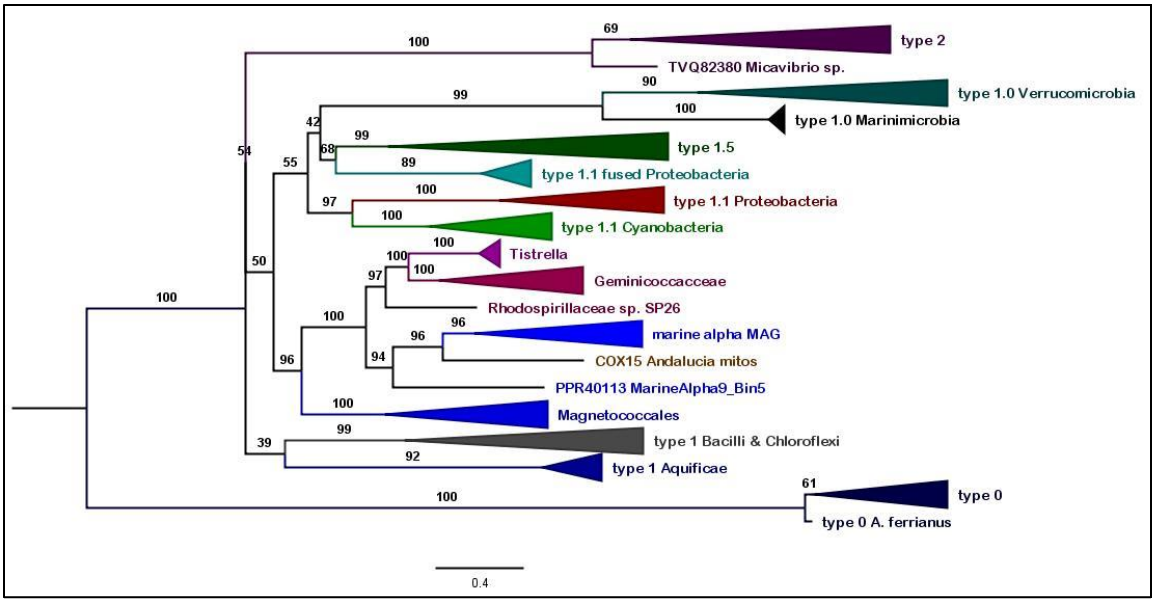

b

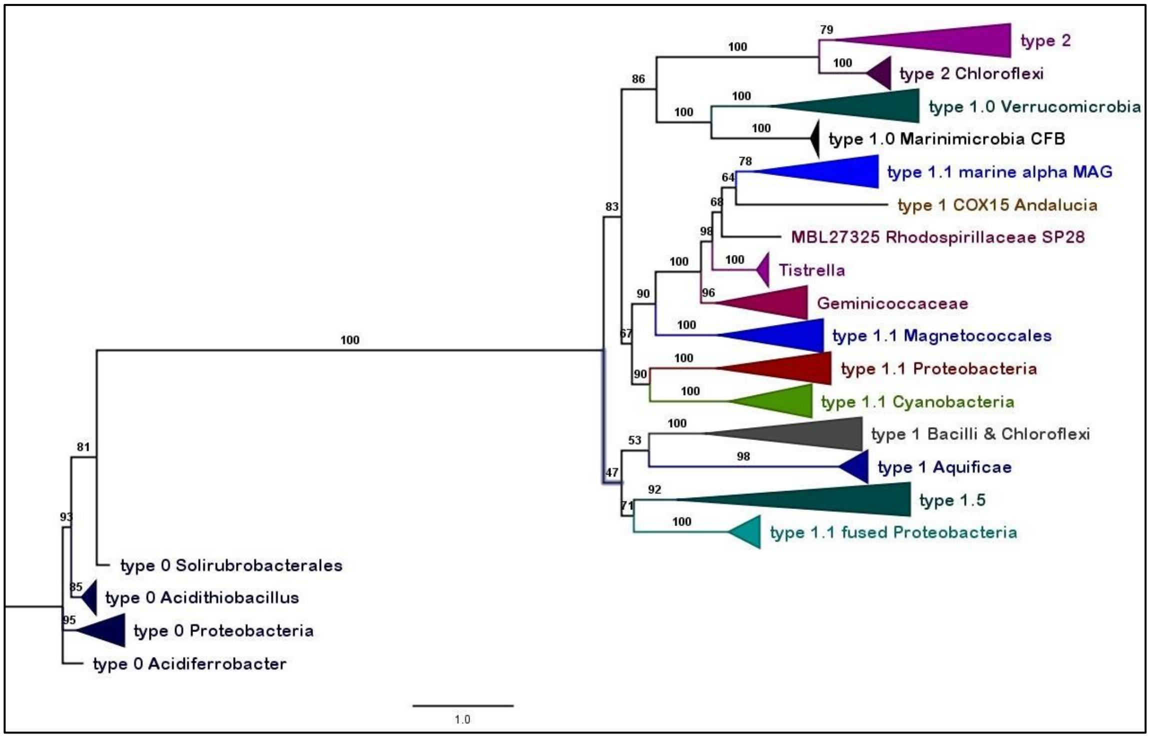

Supplementary Figure S5. **BEAST MCC trees obtained with an alignment of 100 CtaA and DUF420 proteins and statistical analysis of median branch length of several clades.**

Panel a. BEAST MCC and full set of trees obtained with an alignment of 100 CtaA protein sequences. The clade containing proteins of *Alicyclobacillus* that have a significant higher rate of variation is highlighted with a red box. The total number of amino acid sites in the alignment was 363. The mirror image of the cumulative view of the same Bayesian trees obtained with the Densitree program is shown on the right. **Panel b**. Quantitative evaluation of median branch length for selected subclades or (sub)types of CtaA from four separate BEAST MCC trees including that in a, which were built from the alignment of 52 to 100 CtaA proteins (cf. Supplementary Figure S4a). Error bars indicate standard deviation, S.D. The asterisks indicate statistically significant deviations from the values of the subclades on the left (p = 0.03, Mann-Whitney test).

a

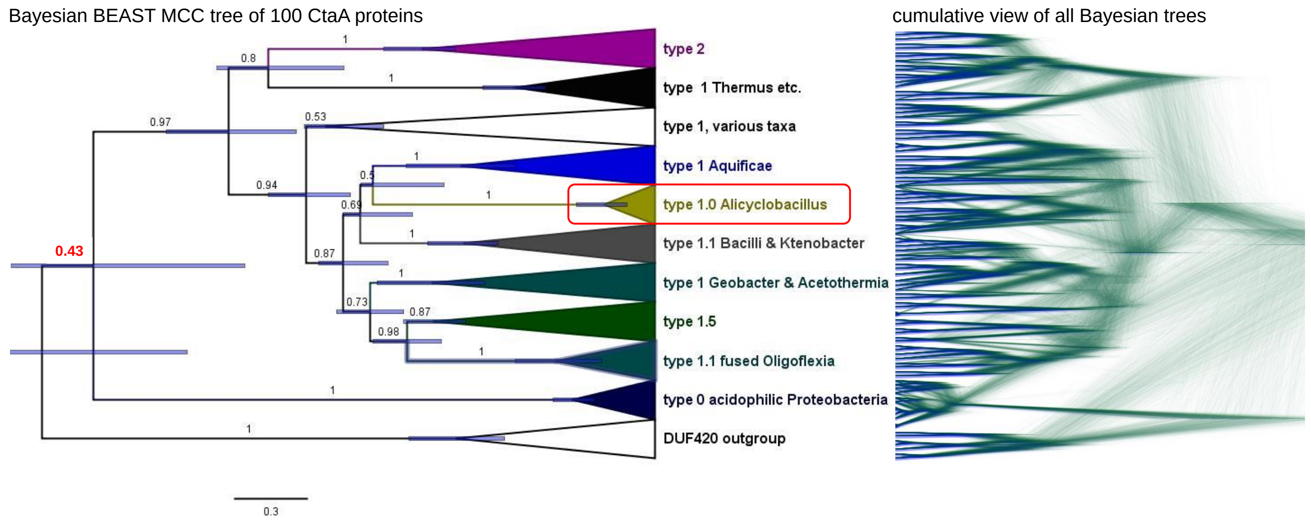

b

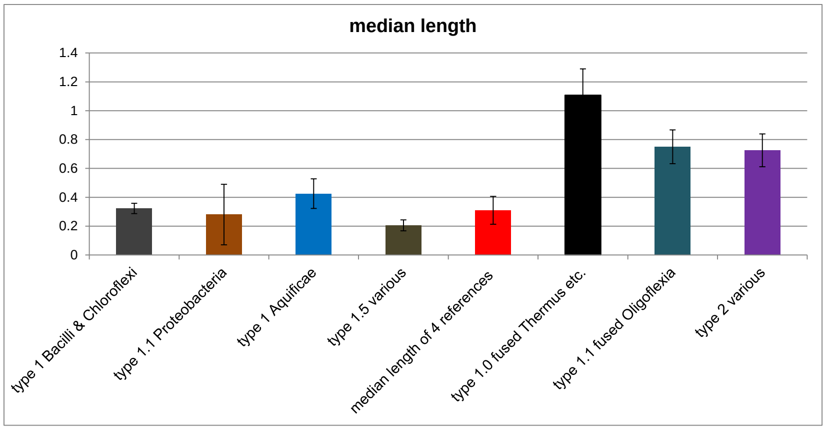

Supplementary Figure S6. Taxonomic distribution of type 0 CtaA.

The NJ tree was obtained from a PSI-BLAST search using *A. ferrooxidans* ACK78561 type 0 CtaA as a query against the version of the nr database (https://blast.ncbi.nlm.nih.gov/Blast.cgi) accessed on 5 September 2020. The search retrieved 118 hits for significant protein orthologs whether it was extended to 250 or more taxa; 28 of these hits were removed because they were either duplicate or partial proteins. Taxa of thermoacidophilic archaea are in light brown as in Figure 5, while acidophilic Proteobacteria are in dark blue; other taxa of Rhodobacterales and Gammaproteobacteria are in red and marine blue, respectively. The outgroup is represented by HHM71647, a type 1.0 CtaA from *Ca.* Fraserbacteria MAG from a hot spring metagenome that was picked by the search with E value worse than threshold, but showing 27% identity with the query. The tree topology is very similar to that obtained earlier from a comparable Blast search (Degli Esposti et al, 2020).

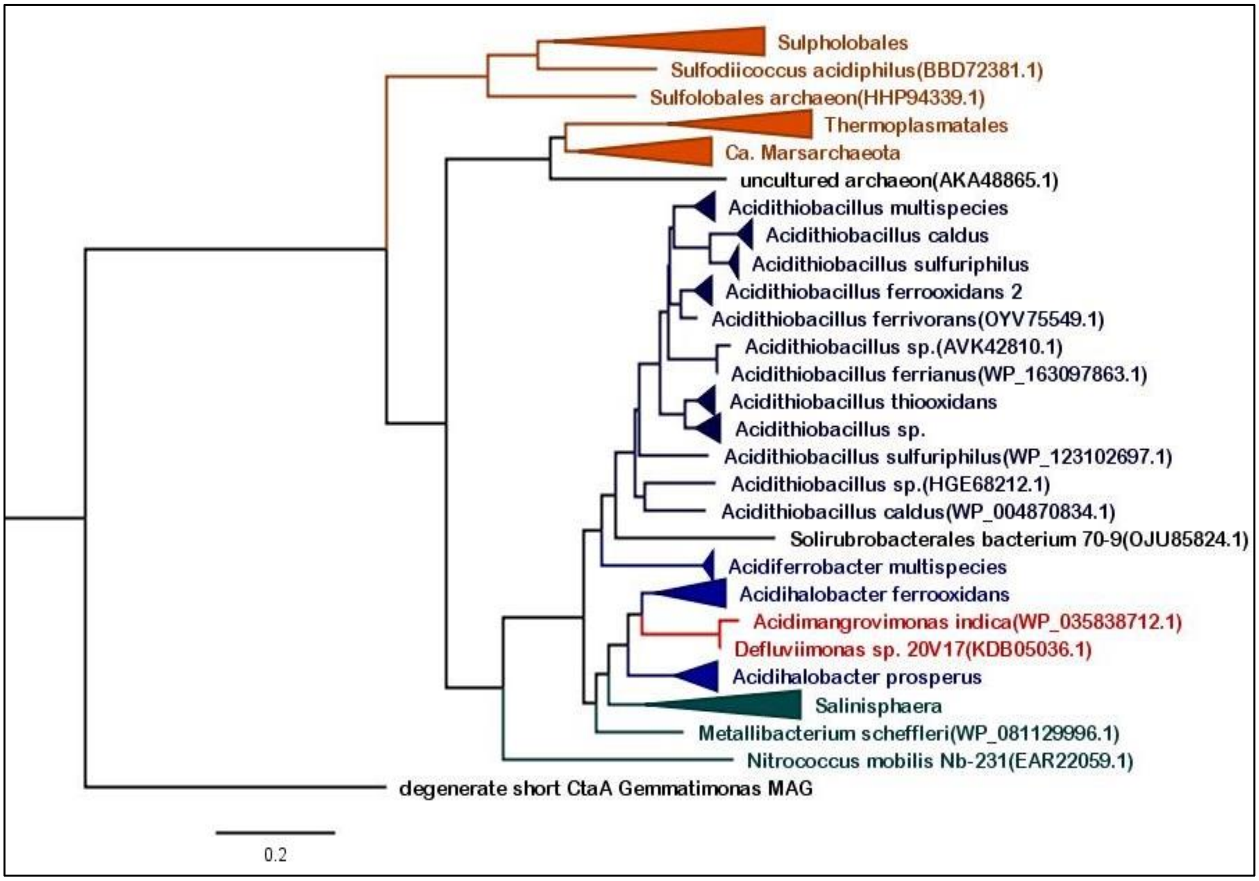

Supplementary Figure S7. Panel a. Amino acid variation of the K-channel in bacterial COX1 and COX2.

			K	K	both	both	both	KC	KC	KC	KC			
Vibrio cholerae **CcoN** numbering	KC-channel	G	M	G	S287	N293	Y227	S240	S244	H247	Y255	COX2		
Paracoccus deni. **COX1** numbering	K-channel	Y280	S291	K354	T351	S357	E292	G	A	M	G	E78	classification	accession
taxon	COX type/subtype													
**C family**														
Leptospirillum spp.	FixN	G	A	F	S	N	Y	S	A	N	Y		KC-channel	WP_014961552
Thioclava_F28-4	FixN with 2TM extra	G	M	G	S	N	Y	S	S	H	Y		KC-channel	WP_078604652
Defluviimonas denitrificans	FixN with 2TM extra	G	M	G	S	N	Y	S	S	H	Y		KC-channel	KDB02090
Rhizobium etli	FixN with 2TM extra	G	M	G	S	N	Y	S	S	S	Y		KC-channel	WP_074061279
Bradyrhizobium japonicum	FixN with 2TM extra	G	M	G	S	N	Y	S	S	H	Y		KC-channel	WP_041955900
Pseudomonas stutzeri 3D	FixN	G	M	G	S	N	Y	S	S	H	Y		KC-channel	5DJQ_A
Mariprofundus sp. EBB-1	FixN	G	T	Y	A	N	Y	S	G	H	Y		hybrid	WP_121542202
**A family**	**COX1**											**COX2**		
Levilinea saccharolytica 2	A2 Anaerolinae	Y	T	K	T	S	E	G	V	S	G	E	K-channel	WP_062417928
Chloroflexi bacterium DOLJORAL78_50_32	A2 2TM Anaerolinae	Y	T	K	T	S	E	G	I	S	G	E	K-channel	PIE82014
Ca. Promineofilum breve	A2 2TM Anaerolinae	Y	T	K	T	S	E	G	V	S	G	E	K-channel	CUS05278
Solirubrobacterales bacterium 70-9	A2 2TM	Y	S	K	T	S	E	G	M	S	S	E	K-channel	OJU85824
Solirubrobacterales bacterium 67-14	A2 2TM	Y	S	K	T	S	E	G	M	A	S	E	K-channel	OJU93486
Chloroflexi bacterium isolate CF_167	A2 1TM hybrid	Y	L	V	T	S	E	A	A	S	S	Q	KC-channel	TMB58219
Chloroflexi bacterium CSP1-4	A2 2TM hybrid	Y	L	V	T	S	E	A	A	S	S	E	KC-channel	KRT60364
Actinobacteria bacterium 15TR583	A2	Y	T	I	T	V	E	G	S	G	S	Q	hybrid	WP_145850914
Acidimicrobium ferrooxidans 1	A2	Y	M	L	T	V	E	A	S	G	S	Q	KC-channel	WP_015799102
Acidithrix ferrooxidans	A2	Y	A	I	T	V	E	G	S	G	S	Q	KC-channel	WP_052605977
Acidimicrobiaceae bacterium USS-CCA1	A2	Y	A	I	T	V	E	G	S	G	S	Q	KC-channel	MST34256
Ktenobacter sp. isolate UBA10483	A2	Y	A	F	T	V	E	G	L	G	S	Q	hybrid	HCJ35412
Dictyobacter aurantiacus	A2	Y	A	F	T	V	E	G	M	S	S	E	KC-channel	WP_126597645
Pseudonocardiales isolate Rrmetagenome	A2	Y	M	L	T	I	E	G	A	S	S	T	KC-channel	PZS18933
Candidatus Dormibacter sp. RRmetagenome_b	A2	Y	M	V	T	V	E	G	A	G	S	A	modified	PZR78255
Chloroflexi bacterium isolate CF_76	A2	Y	M	I	T	V	E	G	G	G	S	I	modified	TME48428
Thermaerobacter marianensis	A2	Y	L	F	T	A	E	G	A	G	S	W	modified	WP_013496006
Thioclava sp. sp. IC9	A2	Y	L	M	T	A	E	G	A	G	S	I	modified	WP_078520553
Thioclava sp. F28-4	A2	Y	L	M	T	A	E	G	A	G	S	I	modified	WP_088728307
Alicyclobacillus ferrooxidans 1	A2 2TM	Y	M	A	T	V	E	G	G	G	S	V	modified	WP_054970767
Sulfobacillus thermosulfidooxidans	A2 2TM	Y	N	A	T	S	E	G	A	G	S	V	hybrid	PSR37978
Acidibacillus ferrooxidans	A2 2TM	Y	Q	A	T	V	E	G	A	G	S	I	hybrid	WP_079290153
Acidiferrobacter thiooxydans	A2 rus operon	Y	L	V	T	A	E	G	A	G	G	V	modified	WP_065972084
Acidiferrobacter sp. SPIII_3	A2 rus operon	Y	L	V	T	A	E	G	A	G	G	V	modified	WP_110137324
Acidithiobacillus ferridurans	A2 rus operon	Y	L	I	T	A	E	G	A	G	G	V	modified	CAA07035
Acidihalobacter ferrooxidans	A2 rus operon	Y	L	M	T	A	E	G	A	G	S	W	modified	WP_083699744
Acidihalobacter prosperus	A2 rus operon	Y	L	F	T	A	E	G	A	G	S	T	hybrid	AOV18356.1
Gloeobacter violaceus	A2	Y	S	K	T	S	E	G	I	S	G	E	K-channel	WP_011142160
Gloeomargarita lithotropha	A2	Y	S	K	T	S	E	G	I	S	G	E	K-channel	WP_071453463
**A2 subtype CyoCAB**														
Sulfuritalea hydrogenivorans	A2 subtype CyoCAB	Y	Y	M	V	G	E	N	V	G	A	A	modified	WP_052473730
Gammaproteobacteria bacterium HyVt-464	A2 subtype CyoCAB	Y	F	M	F	S	E	N	L	A	G	M	modified	HHM04450.1
Rhodocyclaceae bacterium isolate SB9	A2 subtype CyoCAB	Y	F	T	V	G	E	N	V	G	S	A	hybrid	KAB2938899
Magnetovibrio blakemorei	A2 subtype CyoCAB	Y	T	L	V	S	E	N	A	P	G	S	hybrid	WP_069957977
Magnetovspira sp. QH2	A2 subtype CyoCAB	Y	F	L	V	S	E	N	C	G	S	A	KC-channel	WP_046021971
Mariprofundis micogutta zeta	A2 subtype CyoCAB	Y	F	L	F	S	E	N	T	S	S	K	KC-channel	WP_083530385
Deltaproteobacteria bacterium GWA2_55_10	A2 subtype CyoCAB	Y	Y	L	F	A	E	N	T	Y	S	S	KC-channel	OGP15906
Thiotrichaceae bacterium UWMA-0225	A2 subtype CyoCAB	Y	F	M	V	G	E	N	V	G	S	A	KC-channel	HIE02965
Ca. Magnetobacterium casensis	A2 subtype CyoCAB	Y	T	K	T	N	D	G	M	A	G	E	K-channel	WP_040336275
Hydrogenobacter Aquificae	A2 subtype CyoCAB	Y	S	K	T	N	E	G	T	A	G	E	K-channel	WP_096602706
**A2 subtype delta**														
Ca. Sulfobium Nitrospirae	A2 subtype delta	Y	S	K	S	N	E	G	V	S	G	E	K-channel	SPQ00724
Geovibrio	A2 subtype delta	Y	S	K	S	N	E	G	I	S	G	E	K-channel	WP_022851262
Geobacter metallireducens	A2 subtype delta	Y	S	K	S	N	E	G	I	S	G	E	K-channel	WP_004513565
Desulfovibrio vulgaris	A2 subtype delta	Y	S	K	S	N	D	G	I	S	G	E	K-channel	WP_010939102
Melioribacter Ignavibacteria	A2 subtype delta	Y	T	K	S	N	E	G	I	S	G	E	K-channel	WP_014854719
**A2 subtype a-I**														
Nannocystis Myxobacteria	A2 subtype a-I	Y	T	K	S	N	E	G	I	S	S	E	K-channel	WP_096331974
Caldithrix abyssi	A2 subtype a-I	Y	T	K	S	N	E	G	I	S	S	E	K-channel	EHO39949
Bdellovibrio exovorus JSS	A2 subtype a-I	Y	T	K	T	N	E	G	I	S	S	E	K-channel	AGH94669
Tistrella mobilis	A2 subtype a-I	Y	T	K	S	N	E	G	I	S	G	E	K-channel	WP_014744997
Gemmatimonadetes J002	A2 subtype a-I	Y	T	K	S	N	E	G	I	S	G	E	K-channel	RMH73828
Proteobacteria_ isolate ZC4RG46	A2 subtype a-I	Y	T	K	S	N	E	S	I	S	G	E	K-channel	PZN27055
**A1 various subtypes**	corrected													
Elusimicrobia bacterium GWA2_38_7	A1	Y	A	I	S	C	E	G	I	S	S	T	KC-channel	OGR57075
Elusimicrobia bacterium MAG13	A1	Y	S	I	S	C	E	G	I	S	S	E	hybrid	NNN06642
bacterium HR19 (Calescamantes)	A1 simple operon	Y	F	T	F	S	E	S	V	A	S	I	KC-channel	GBD04318
Methylacidiphilum sp. Yel	A1	Y	G	L	S	V	E	S	F	S	S	E	KC-channel	WP_134389983
Halobacteriovorax multispecies	A1	Y	S	F	S	N	D	G	T	S	S	I	KC-channel	WP_114706215
Bacteriovorax stolpii	A1	Y	S	F	S	N	D	G	T	A	S	A	hybrid	WP_102242033
Ca. *Entotheonella gemina*	A1	Y	S	K	S	N	E	G	M	S	S	E	hybrid	ETX06475
Acidobacteria isolate gp22_AA2	A2 type	Y	T	K	S	N	E	G	I	S	G	E	K-channel	PYS96483
Acidobacteria bacterium isolate gp2 AA90	A1 type	Y	T	K	S	N	D	G	G	S	G	L	K-channel	PYT95786
Acidobacteria_13_1_40CM_2_68_5	A2 type	Y	T	K	S	N	D	G	I	S	G	E	K-channel	OLD64351
Chloroflexi_soil_metagenome3	A2 type, new operon	Y	T	K	S	N	E	G	I	S	G	E	K-channel	TMG33390
Gemmatimonadetes_AG37	A1 type	Y	T	K	S	N	D	G	I	A	G	E	K-channel	PYO98997
bacterium HR33 Gemmatimonadetes	A1	Y	S	K	S	N	E	G	A	G	G	E	K-channel	GBD31927
Chlorobi bacterium isolate J074	A1	Y	S	K	S	N	E	G	V	S	S	E	K-channel	RMF33953
Sphingobacterium detergens	A1 a-III subtype split	Y	S	K	S	N	E	G	M	S	S	E	K-channel	RKE44380
Flavobacterium johnsonii	A1 a-III subtype split	Y	S	K	S	N	E	G	M	S	S	E	K-channel	WP_073407898
Burkholderia multispecies	A1 a-III subtype	Y	S	K	T	N	D	G	G	A	S	E	K-channel	WP_007741208
Methylocystis sp. SC2	A1 a-III subtype	Y	S	K	T	N	D	G	G	A	S	E	K-channel	WP_014891915
Bacillus subtilis	A1 caa3	Y	S	K	T	N	E	G	M	A	G	E	K-channel	WP_041905325
Staphylococcus multispecies	A1 caa3	Y	S	K	T	N	E	G	M	A	G	E	K-channel	WP_078356817
Ardenticatena maritima Chloroflexi	A1	Y	S	K	T	N	E	G	V	A	G	E	K-channel	WP_054492930
Defluviimonas sp. 20V17	A1 bo3 subtype	Y	S	K	T	D	E	G	L	A	S	E	K-channel	KDB01762
Acidithiobacillus caldus	A1 bo3 subtype	Y	S	K	T	N	E	G	M	A	S	E	K-channel	WP_004870827
Acidithiobacillus sulfuriphilus	A1 bo3 subtype	Y	S	K	T	N	E	G	M	A	S	E	K-channel	WP_123101886
Acidithiobacillus thiooxidans	A1 bo3 subtype	Y	S	K	T	N	E	G	M	A	S	E	K-channel	WP_010643015
Nitrococcus mobilis	A1 subtype ab	Y	S	K	T	N	Q	G	M	A	S	E	K-channel	WP_005000539
Salinisphaera halophila	A1 subtype ab	Y	S	K	T	N	T	G	M	A	S	E	K-channel	WP_123590938
Defluviimonas sp. 20V17	A1 subtype b	Y	S	K	T	S	H	G	M	A	G	E	K-channel	KDB02090
Paracoccus denitrificans 3D	A1 subtype b	Y	S	K	T	S	H	G	M	A	G	E	K-channel	3EHB_A
Rhodobacter sphaeroides 3D	A1 subtype b	Y	S	K	T	S	H	G	M	A	G	E	K-channel	1M57_A
Tistrella mobilis	A1 subtype b	Y	S	K	T	S	H	G	M	A	G	E	K-channel	WP_041604984

cont.

Supplementary Figure S7. Panel b. Amino acid variation of the K-channel in archaeal COX1 and COX2.

Vibrio cholerae **CcoN** numbering	**KC-channel**	G	M	G	S287	N293	Y227	S240	**S244**	**H247**	Y255			
Paracoccus deni. **COX1** numbering	**K-channel**	Y280	S291	**K354**	T351	S357	E292	G	A	M	G	**E78**	**classification**	**accession**
	**COX1**											**COX2**		
Haloarcula COX13?														
Halobacterium salinarum Euryarchaeota	A1 type	**Y**	S	K	S	N	L	G	V	S	S	E	**K-channel**	WP_010902450
Ca. Heimdallarchaeota_LC_3	A1 type not fused	**Y**	S	K	S	N	L	G	M	S	G	E	**K-channel**	OLS27277
Ferroplasma acidiphilum Thermoplasmatales	A1 type COX13	**Y**	F	L	T	G	D	L	M	I	A	F	**modified**	WP_171481686
Picrophilus torridus Thermoplasmatales	A1 type COX13	**Y**	F	L	T	G	D	I	M	I	A	F	**modified**	WP_153274183
Ca. Marsarchaeota strains	A1 type COX13	**Y**	A	M	F	D	E	Y	A	L	L	L	**modified**	PSN98237
Aeropyrum camini Chenarchaeota	A1 type COX13	**Y**	A	A	F	S	D	A	I	A	S	L	**modified**	WP_158318587
Haloarcula hispanica	A1 type COX13	**Y**	A	V	S	S	E	G	V	S	S	F	**hybrid**	WP_151103863
Pyrobaculum Crenarchaeota	A1 type COX13	**Y**	S	K	N	N	E	A	I	S	S	G	**hybrid**	AAL63417
Sulfolobus acidocaldarious	SoxM A1 type COX13	**Y**	S	K	S	N	E	G	I	S	S	L	**hybrid**	WP_011279051
Sulfolobus & other Sulfolobaceae	SoxM A1 type COX13	**Y**	G	K	S	S	E	G	I	S	S	K	**K-channel**	BBD73009
Thermoplasmatales archaeon A-plasma	bb(o)3 hybrid with B	**Y**	Y	T	S	N	D	S	A	S	S	E	**K-channel**	EQB72844
Cuniculoplasma divulgatum	bb(o)3 hybrid with B	**Y**	Y	T	S	N	E	S	G	G	S	E	**K-channel**	WP_077076430
Haloarcula & other Euryarchaeota	**ba3 classical**	**Y**	Y	T	S	T	T	S	A	V	S	E	**K-channel**	WP_004965245

The original excel file from which this figure has been built is supplied as: Supplementary Table for Fig. S7.

Figure S8. Extended phylogenic tree of COX1 from bacterial and archaeal taxa.

Panel a. The ML tree was obtained with the program IQ-Tree using an alignment of 100 COX1 protein sequences from family A and B oxidases that covers the majority of the subclades analyzed here (Supplementary Table S1 – see also Fig. 6). The mixture model of amino acid substitution EX_EHO (Le et al, 2010) was used for a total of 600 amino acid sites. Trees with essentially the same topology were obtained using the LG model (Fig. 6), but with less robust support in some internal nodes, expressed in %Ultrafast bootstraps as indicated. The few values ≤ 85% are shown, with the single one below 50% in red. The proteins were selected from diverse taxa as to represent the majority of COX operons that are currently recognized in bacteria lineages (Supplementary Table S1, cf. Degli Esposti, 2020) and were aligned with a combination of computer-assisted and manual refinement to match the known structure of *P. denitrificans* COX, as previously described (Pereira et al, 2001: Degli Esposti et al, 2020). The alignment, available upon request, included four FoxA paralogs from iron-oxidizing Sulfolobaceae (Kozubal et al., 2011) and was trimmed at the N terminus as in previous studies (Degli Esposti et al, 2019; Degli Esposti et al, 2020; Kozubal et al, 2011; Matheus et al, 2019; Sousa et al, 2012; Spang et al, 2019). The fully expanded version of the tree showing the accession of each protein is supplied as a separate .pdf file named: COX1refined100IQTreeEX_EHOFigTreefull. **Panel b**. Statistical analysis of the branch length of selected subclades of COX1 from n = 5 ML trees reconstructed with the IQ-Tree program as in panel a and also with the LG and WAG model of amino acid substitution. Error bars represents Standard Deviation values (SD); the three columns spaced on the right are statistically different from that of either subtype (a)b or Cyanobacteria in the left (p = 0.011, Mann-Whitney test).

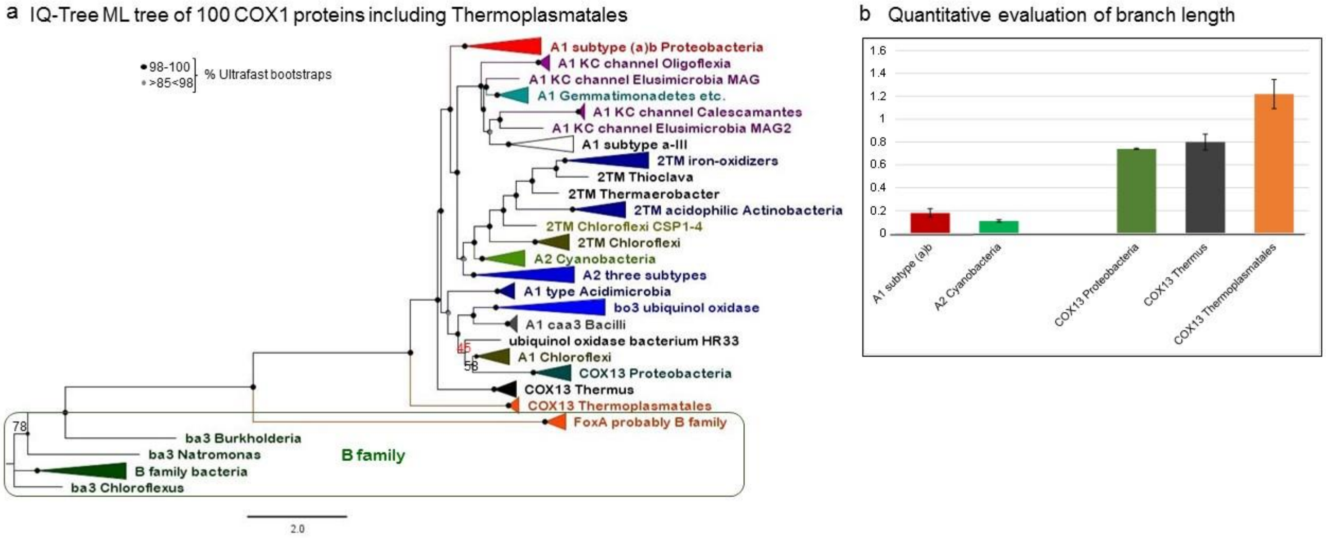

Figure S9. **Phylogeny of bacterial COX1.**

Panel a. Bayesian tree with annotated ancestral features. The Bayesian BEAST MCC tree was obtained with an alignment of 74 bacterial COX1 sequences and 6 FixN sequences, with a total of 682 amino acid sites. The distribution of variants of the K-channel and extra TM at the N terminus is annotated as in Figure 5. The values of posterior support was generally 100% except for the four branches with annotated numbers. Note the weak support (47%, in red as in Supplementary Figure S1) for the bottom clade combining A2 type with 2TM proteins. **Panel b**. Median rate of substitution of selected subclades of A1 type COX1 was calculated from the values obtained from *n* = 5 separated Bayesian BEAST MCC trees as in Panel a, which included different combination of 64 to 90 COX1 proteins. The rate value for the *bo*3 ubiquinol oxidase is at least 3-fold larger than that of other clades (p = 0.012, Mann-Whitney test).

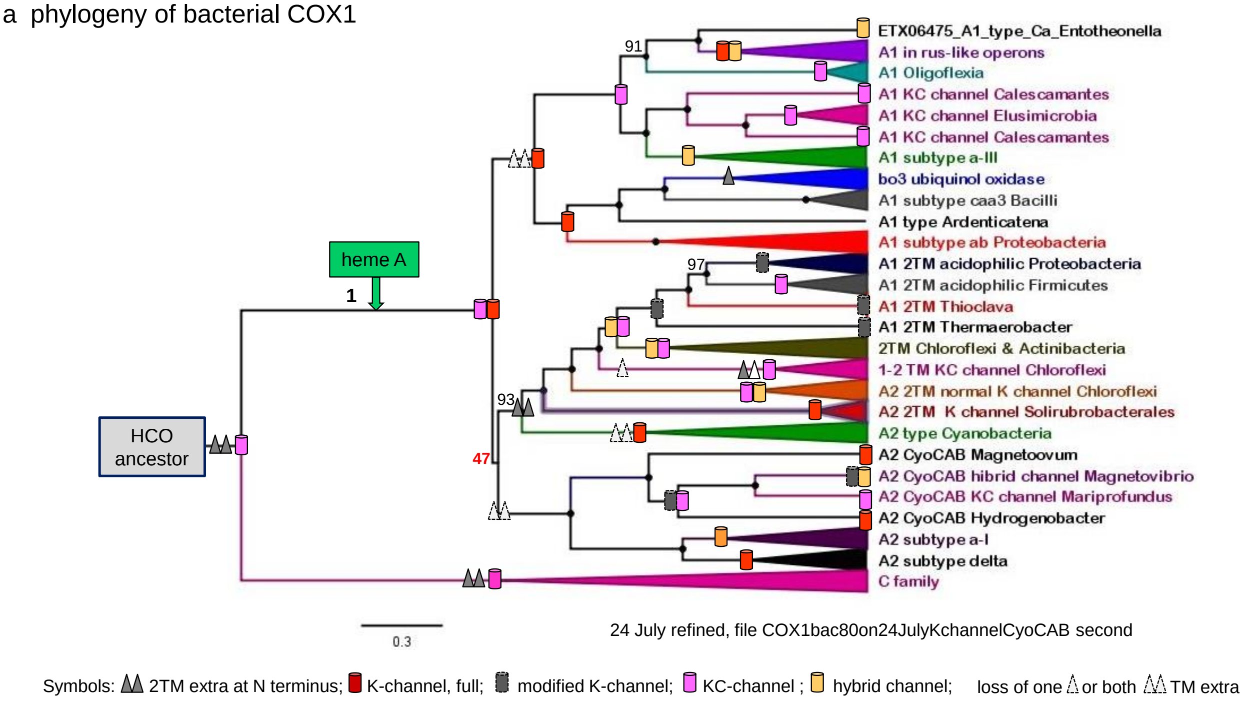

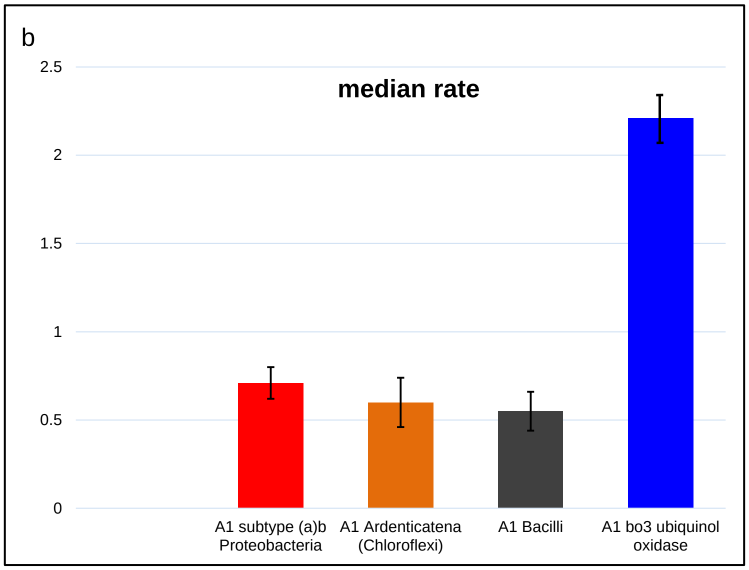

Figure S9 panel c. The representative ML tree was obtained with the program PhyML 3.0 using an alignment of 78 bacterial COX1 and FixN protein sequences that included their N terminus as in Figure 7, with a total of 682 amino acid sites. SH-like support is shown only for the nodes displaying less than 90% values. The distribution of the 2TM extra feature and of the different variants of the K-channel classified in Supplementary Figure S7 are annotated onto the tree branches using the symbols shown at the bottom of the figure, as in Figure 5. No significant change in the composition of the D-channel was observed among the proteins analyzed here.

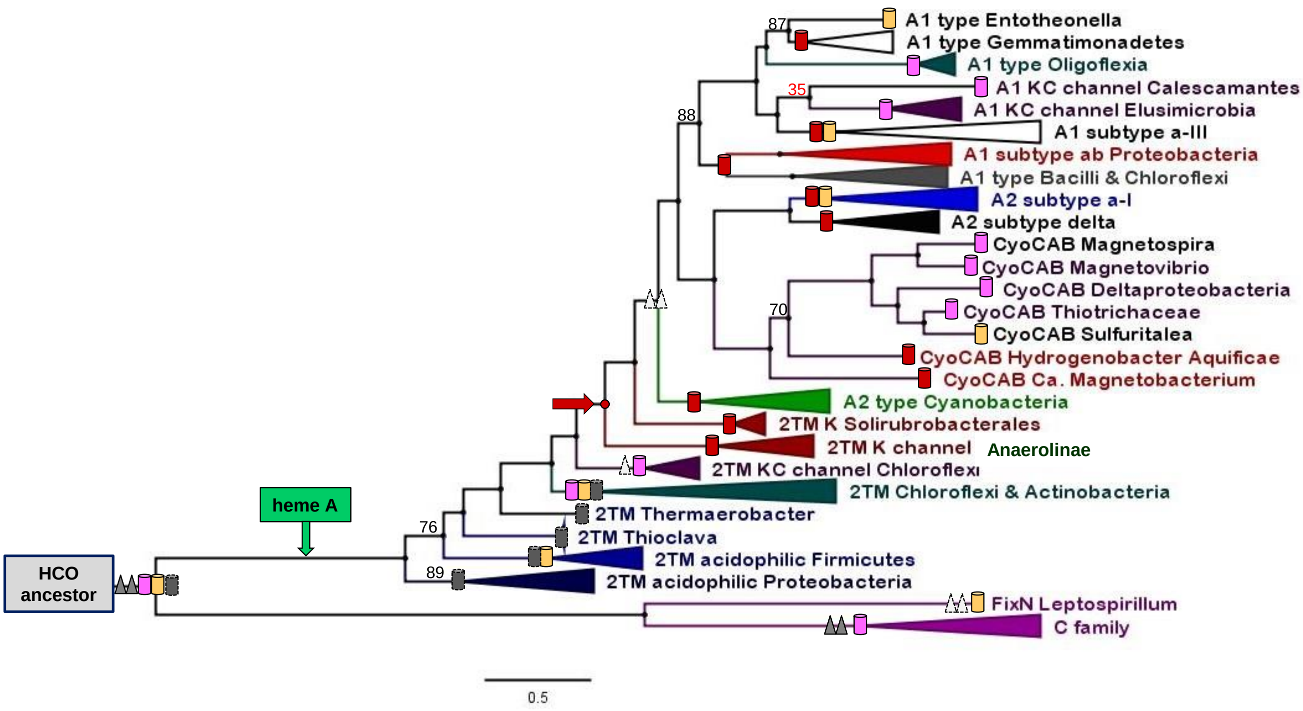

Symbols: 2TM extra at N terminus; K-channel, full; modified K-channel; KC-channel ; hybrid channel; loss of one or both TM extra

Supplementary Figure S10. ML trees of 22 COX1 and 2 FixN sequences.

ML trees of the new alignment of 22 COX1 protein sequences and two FixN sequences to build a solid COX1 phylogeny. The alignment had a total of 651 amino acid sites.

a MEGA ML tree of 24 COX1 proteins

b PhyML ML tree of 24 COX1 proteins

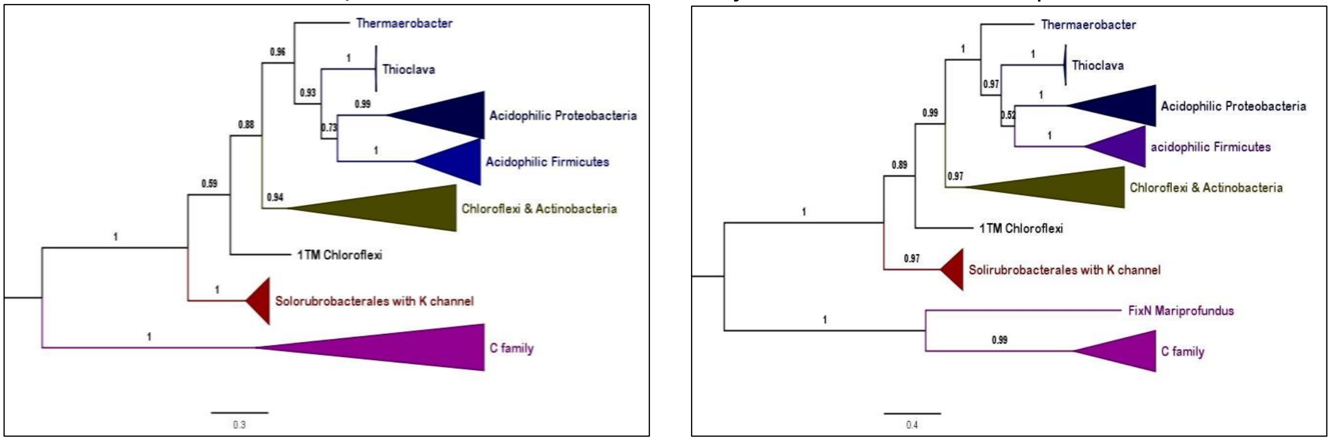

Supplementary Figure S11. Branch length evaluation in a robust Bayesian tree of 48 COX1 proteins.

Panel a. Bayesian BEAST MCC tree with annotated median values of branch length. The alignment had a total of 651 amino acid sites. Black circles indicate posterior support >0.9. **Panel b**. Histogram plot of the range in 95%_HPD values (High Posterior Density, equivalent to confidence range) for the subclades of COX1 proteins in the tree shown in Panel a (see also Supplementary Table S1). The top part of the histogram is coloured in pink when the bottom value in 95%_HPD branch length intervals clearly exceeds the top value in the reference clade of A1 subtype b and/or is higher than the median of the lower 95%_HPD values plus 3SD of all other subclades (see section on statistical analysis in Supplementary Material). The red dashed line indicates such a reference median plus 3SD value.

a Bayesian BEAST MCC tree of 48 COX1 proteins with median branch length

b Range of branch length of COX1 subclades

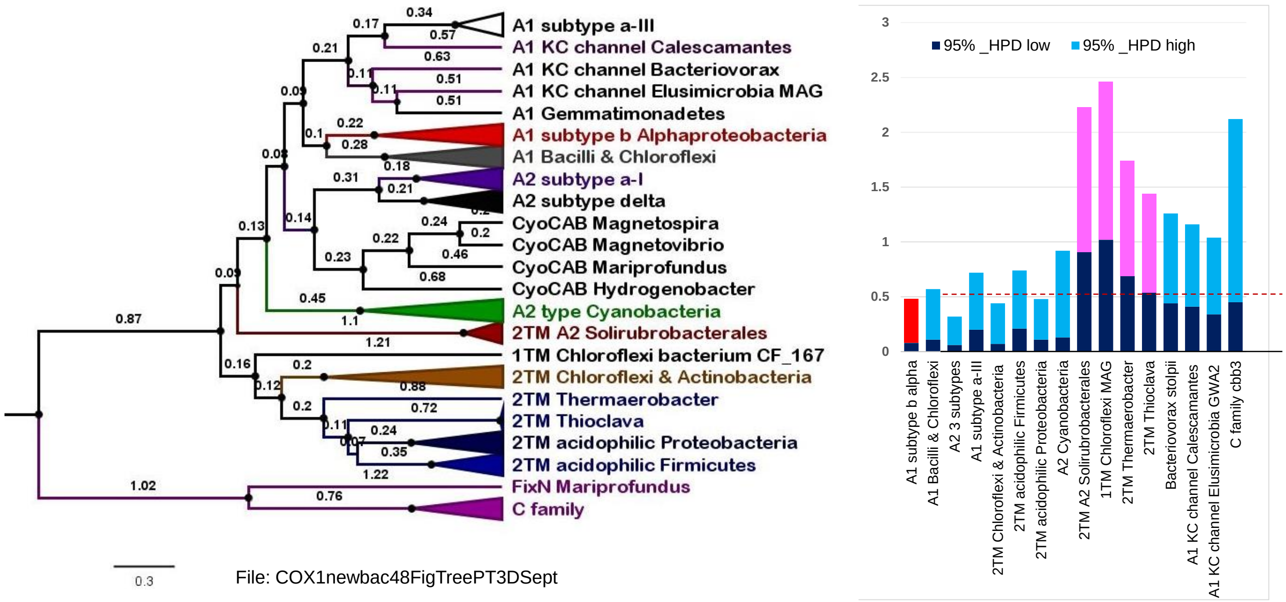

Supplementary Figure S12.

Panel a. Bayesian BEAST MCC tree with 40 COX1 protein sequences including two proteins of the A1 type subclade a-III that show a branch length longer than average. The alignment had a total of 651 amino acid sites as in Supplementary Figure S10. The orange circle surrounds the node with low support to these proteins. **Panel b**. DensiTree image of the same 1801 Bayesian tree in panel a. **Panel c**. ML tree obtained with the PhyML program using the same alignment of 52 COX1 proteins without outgroup paralogs as that used in Figure 6B.

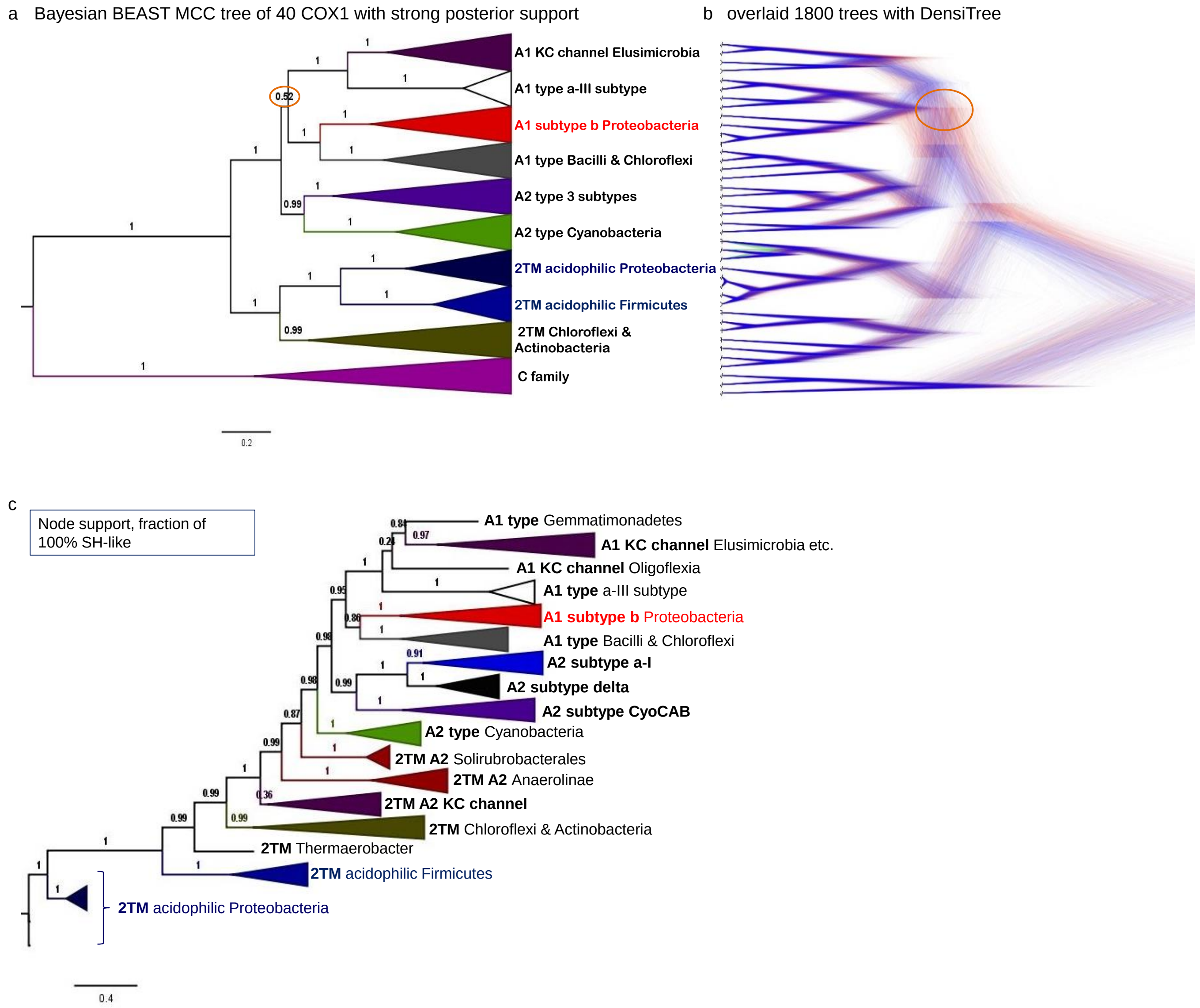

Supplementary Figure S13.

Comparison of ML trees of caa3_CTAG proteins obtained with an alignment that included functionally related MATE proteins as the potential outgroup (**Panel a**, cf. [Degli Esposti et al, 2020] and another alignment without such outgroup proteins (**Panel b**). The alignments had a total of 243 amino acid sites.

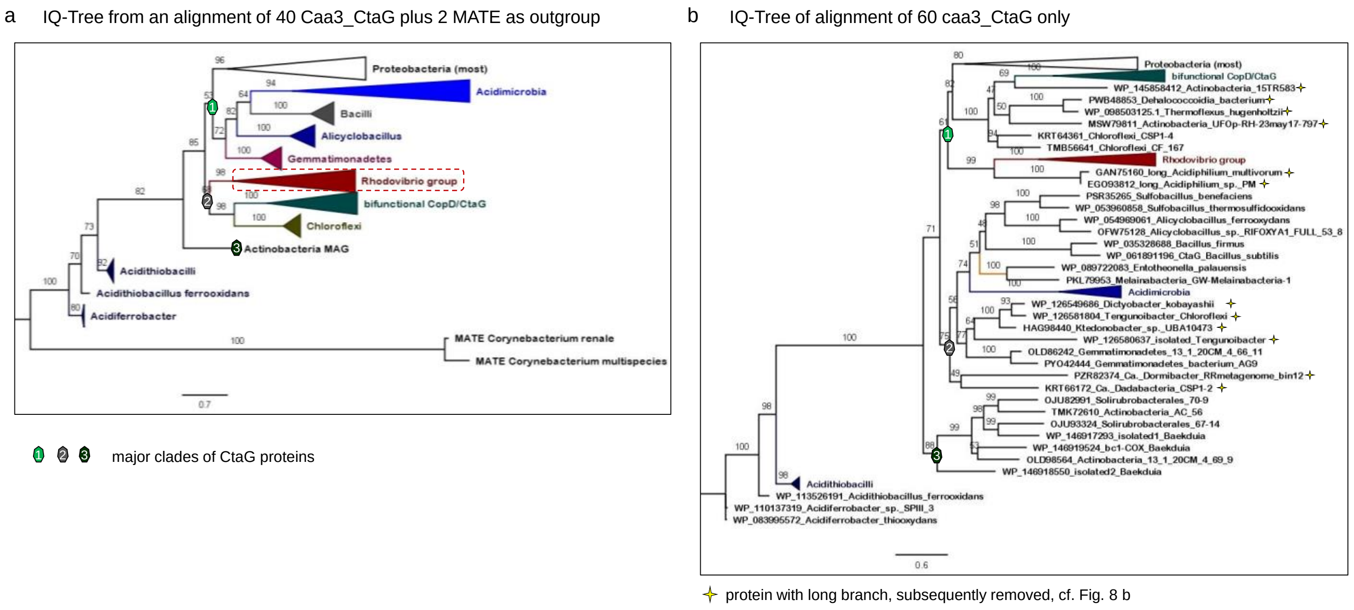

Supplementary Figure S14.

Panel a. Bayesian BEAST tree of 44 caa3_CtaG proteins, some of which retain a long branch (indicated by the cross symbol) and have subsequently been removed to produce the Bayesian tree in Figure 7B.

Panel b. IQ-Tree ML consensus tree of the same alignment of 40 CtaG proteins that has been used for reconstructing the Bayesian tree in Figure 7B. The alignment had a total of 243 amino acid sites.

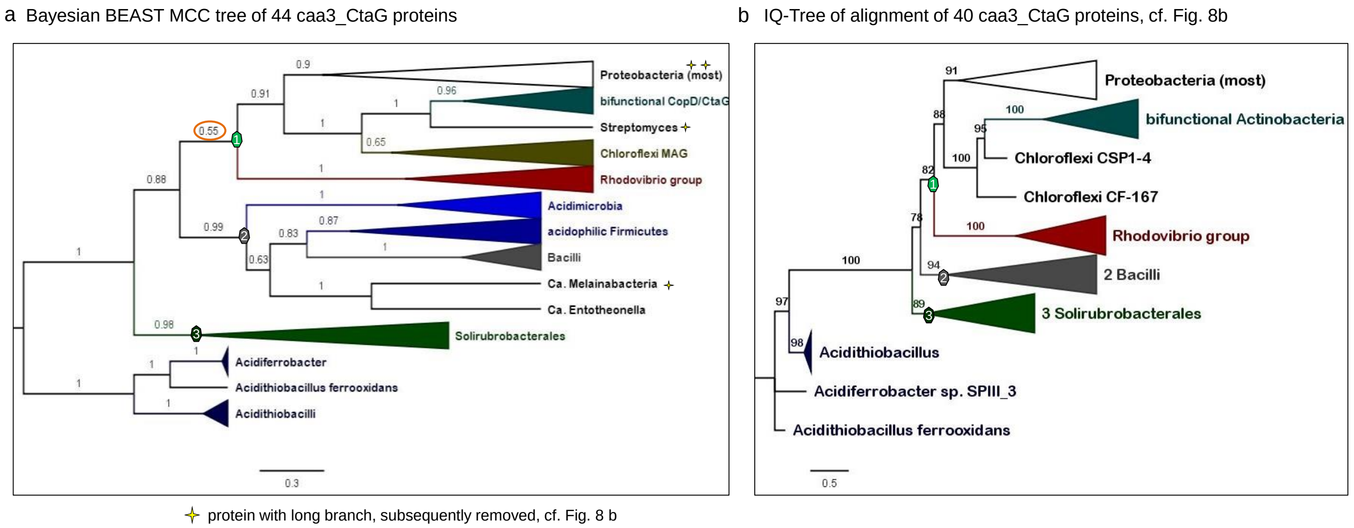

Supplementary Figure S15.

ML tree of an alignment of 46 CtaG_Cox11 sequences showing the likely sister group of bacterial proteins to eukaryotic Cox11 (the eukaryotic clade is highlighted). The sequences included 23 mitochondrial Cox11 from taxa which represent the majority of currently recognized supergroups of eukaryotes (Supplementary Table S2) and two from deep branching Proteobacteria that show a peculiar insert just before the conserved Cys motif of the protein (Banci et al, 2004), e.g. MAF31647 from a Magnetococcales MAG. The tree was obtained with the IQ-Tree program and the EX_EHO mixture model and contained 231 amino acid sites; it is representative of *n*=21 ML trees obtained with different programs and models, using Cox11 alignments spanning 44 to 106 proteins. See Supplementary Table S2 for the accession and specific taxa of the proteins of this tree.

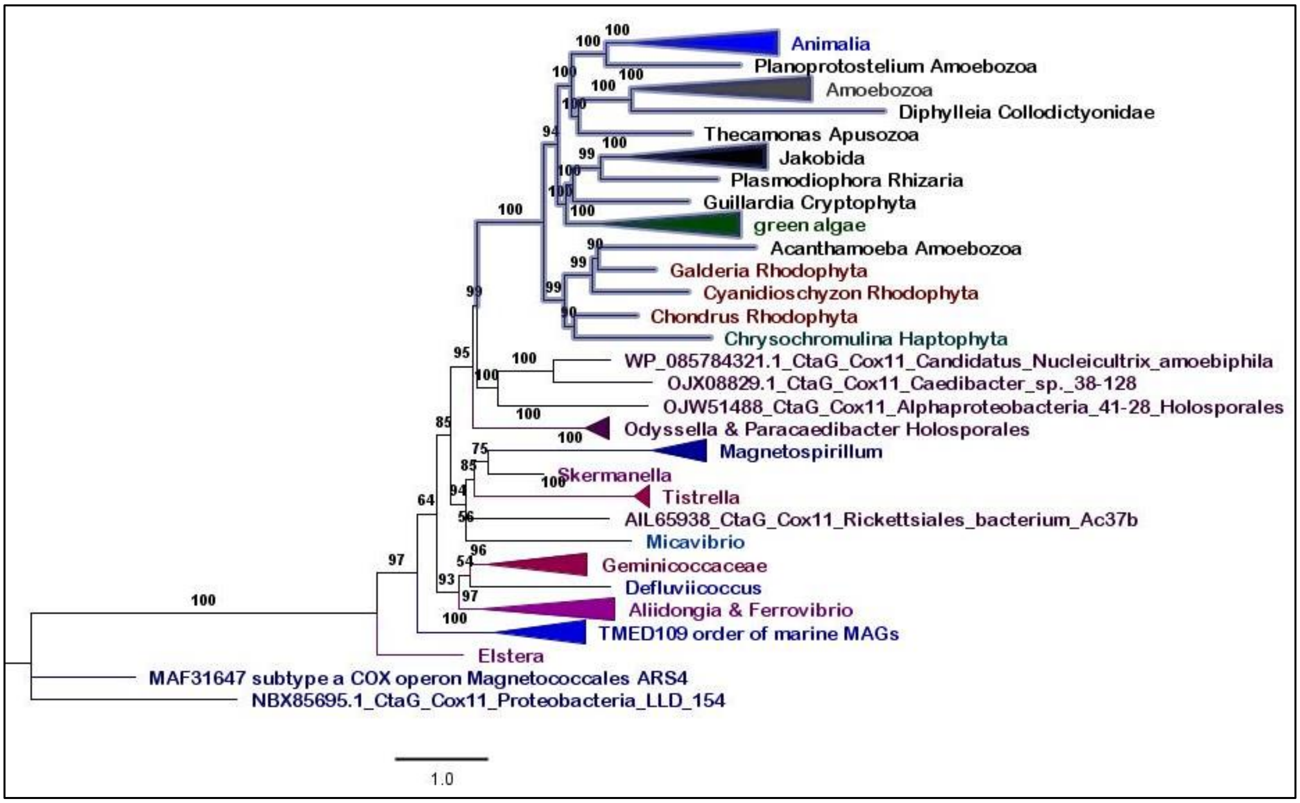

Supplement: Supplementary file 2 [file Data_Sheet_2.pdf]
